# Quantitative Analysis of Cotton Canopy Size in Field Conditions Using a Consumer-Grade RGB-D Camera

**DOI:** 10.3389/fpls.2017.02233

**Published:** 2018-01-30

**Authors:** Yu Jiang, Changying Li, Andrew H. Paterson, Shangpeng Sun, Rui Xu, Jon Robertson

**Affiliations:** ^1^Bio-sensing and Instrumentation Laboratory, School of Electrical and Computer Engineering, College of Engineering, University of Georgia, Athens, GA, United States; ^2^Plant Genome Mapping Laboratory, College of Agricultural and Environmental Sciences, University of Georgia, Athens, GA, United States; ^3^Department of Genetics, Franklin College of Arts and Sciences, University of Georgia, Athens, GA, United States

**Keywords:** cotton, high-throughput, phenotyping, field, RGB-D, morphological

## Abstract

Plant canopy structure can strongly affect crop functions such as yield and stress tolerance, and canopy size is an important aspect of canopy structure. Manual assessment of canopy size is laborious and imprecise, and cannot measure multi-dimensional traits such as projected leaf area and canopy volume. Field-based high throughput phenotyping systems with imaging capabilities can rapidly acquire data about plants in field conditions, making it possible to quantify and monitor plant canopy development. The goal of this study was to develop a 3D imaging approach to quantitatively analyze cotton canopy development in field conditions. A cotton field was planted with 128 plots, including four genotypes of 32 plots each. The field was scanned by GPhenoVision (a customized field-based high throughput phenotyping system) to acquire color and depth images with GPS information in 2016 covering two growth stages: canopy development, and flowering and boll development. A data processing pipeline was developed, consisting of three steps: plot point cloud reconstruction, plant canopy segmentation, and trait extraction. Plot point clouds were reconstructed using color and depth images with GPS information. In colorized point clouds, vegetation was segmented from the background using an excess-green (ExG) color filter, and cotton canopies were further separated from weeds based on height, size, and position information. Static morphological traits were extracted on each day, including univariate traits (maximum and mean canopy height and width, projected canopy area, and concave and convex volumes) and a multivariate trait (cumulative height profile). Growth rates were calculated for univariate static traits, quantifying canopy growth and development. Linear regressions were performed between the traits and fiber yield to identify the best traits and measurement time for yield prediction. The results showed that fiber yield was correlated with static traits after the canopy development stage (*R*^2^ = 0.35–0.71) and growth rates in early canopy development stages (*R*^2^ = 0.29–0.52). Multi-dimensional traits (e.g., projected canopy area and volume) outperformed one-dimensional traits, and the multivariate trait (cumulative height profile) outperformed univariate traits. The proposed approach would be useful for identification of quantitative trait loci (QTLs) controlling canopy size in genetics/genomics studies or for fiber yield prediction in breeding programs and production environments.

## 1. Introduction

Cotton (*Gossypium*) is one of the most important textile fibers in the world, accounting for about 25% of total world textile fiber use (USDA-ERS, 2017). Thus, improvement of cotton production is vital to fulfilling the fiber requirements of over nine billion people by 2050 (Reynolds and Langridge, 2016). Plant canopy structure is an important trait, affecting crop functions such as light-energy production and utilization (Norman and Campbell, 1989). Optimal canopy structure can improve plant photosynthesis and thus crop yield potential (Reta-Sánchez and Fowler, 2002; Stewart et al., 2003; Giunta et al., 2008). One key to increasing yield is to figure out the optimal canopy structure for maximizing plant photosynthesis (Murchie et al., 2009; Zhu et al., 2010). Canopy size is an important aspect of canopy structure and critical to plant photosynthesis, fruiting, and biomass accumulation. However, assessment of canopy size becomes a bottleneck, which limits breeding programs and genetics studies (White et al., 2012; Cobb et al., 2013; Araus and Cairns, 2014; Barabaschi et al., 2016), especially for large crop populations and high-dimensional traits (e.g., canopy volume). Accurate and high throughput techniques for quantifying canopy size would facilitate cotton (and other) breeding programs and genetics studies (Araus and Cairns, 2014; Pauli et al., 2016; Reynolds and Langridge, 2016).

Canopy size is spatially and temporally variable, and morphological traits describing canopy size can be grouped based on different criteria (Norman and Campbell, 1989). From the spatial perspective, component traits of canopy size can be categorized as one-dimensional and multi-dimensional traits. One-dimensional (1D) traits quantify canopy size in a single dimension (e.g., canopy height and width), whereas multi-dimensional traits quantify canopy size by considering multiple dimensions (e.g., canopy area in two-dimensional (2D) space and volume in three-dimensional (3D) space). From the temporal perspective, the traits can be separated into static and dynamic categories. Static traits are directly measured for canopies at a certain time, whereas dynamic traits (e.g., growth rates) are the change of static traits over time. In addition to spatial and temporal criteria, data dimensionality is an important consideration of morphological traits for canopy size quantification. Based on the number of variables, morphological traits can be classified as univariate (e.g., maximum and average canopy height) or multivariate (e.g., cumulative height profile describing heights at different percentiles).

Univariate traits are widely used due to their simplicity (easy to define) and availability (most measurement instruments provide a single reading). One-dimensional traits can be measured manually (or automatically) using distance meters. These 1D traits represent canopy size in only one dimension, creating a potential bias in structure assessment and comparison (Norman and Campbell, 1989). Leaf area as a 2D trait has been widely used for canopy size studies, and its derivative indicator (leaf area index, LAI) has demonstrated successes in estimating crop photosynthetic activities, biomass, yield, and biotic/abiotic stress tolerance (Sinclair and Horie, 1989; Kross et al., 2015; Feng et al., 2017). Leaf area can be directly measured using destructive methods, such as using a leaf area meter or image scanner to obtain the area of leaf samples that have been cut off the plant. Such destructive methods are usually arduous and hard to apply in large-scale experiments. To address those issues, LAI can be indirectly estimated using instruments such as the LI-COR LAI-2200 and Decagon LP-80 (Bréda, 2003; Weiss et al., 2004). Conventional 2D imaging methods can also be applied to calculate projected leaf area or canopy coverage ratio, estimating the true leaf area or LAI (Jonckheere et al., 2004; Zheng and Moskal, 2009). Nonetheless, all aforementioned methods require laborious data collection and do not provide 3D information, and therefore are inadequate to rapidly and comprehensively quantify canopy size and development.

Advanced 3D imaging approaches provide new opportunities to accurately quantify canopy size in multiple dimensions such as canopy height and width (1D), leaf area (2D), and volume (3D). The approaches can be categorized into passive and active 3D imaging (Li et al., 2014).

Passive 3D imaging methods reconstruct 3D structures of objects by expanding conventional 2D imaging methods. Stereo vision and the structure-from-motion (SfM) technique are two representative passive imaging methods. Small unmanned aerial systems (UASs) provide a means to quickly collect images for 3D reconstruction and trait extraction (e.g., crop height) using digital surface models and photogrammetry. However, images collected by UASs usually have lower quality (e.g., image resolution and sharpness) than images from ground systems, which could significantly affect the reconstruction accuracy (Shi et al., 2016) or even cause failures of 3D structure reconstruction (Jay et al., 2015). In addition, most of the passive techniques are computationally expensive (taking several hours for reconstruction of one plot) (Jay et al., 2015; Muller-Linow et al., 2015; Nguyen et al., 2016; Dong et al., 2017). Speeding up the process would require either using high-performance computing (HPC) resources, which would impose a considerable cost for large-scale breeding programs, or limiting the experimental scale and therefore breeding efficiency.

Active 3D imaging methods directly acquire 3D information by operating external light sources. Commonly used sensors include light detection and ranging (LiDAR), triangulation laser scanners, and time-of-flight (TOF) and structured light cameras. With suitable camera setup and illumination, most active methods can accurately obtain 3D point clouds of plants in field conditions for canopy size analysis, but the instruments are usually expensive ($2,000–100k or more) compared with cameras used in passive methods (Li et al., 2014). Recent advances in consumer-grade RGB-D cameras (e.g., Microsoft Kinect and ASUS Xtion) provide an inexpensive solution for 3D scanning (Nock et al., 2013; Paulus et al., 2014; Andujar et al., 2016). In particular, the Microsoft Kinect v2 camera uses the TOF principle with upgraded color and depth resolution, creating the possibility for inexpensive and high-resolution 3D sensing in field conditions. Several previous studies explored the use of Kinect v2 camera in measuring canopy size (height and volume) in field conditions, finding that the Kinect v2 was a promising tool for field-based phenotyping (Andújar et al., 2016; Jiang et al., 2016; Andujar et al., 2017). However, two aspects need to be further improved: (1) data processing should be fully automated to improve throughput, and (2) more spatially (single and multi-dimensional traits) and temporally (static and dynamic traits) morphological traits need to be measured and studied.

The overall goal of the study was to develop a 3D imaging approach to automatically and quantitatively analyze canopy size of cotton plants in field conditions. Specific objectives were to (1) develop algorithms to reconstruct colorized point clouds of individual plots using depth and color images collected by Kinect v2 camera, (2) develop algorithms to segment canopy point clouds from the ground and weeds, (3) extract canopy size traits (canopy height, width, projected area, and volume) and their dynamic changes over time, and (4) explore the potential of using the extracted traits for fiber yield prediction.

## 2. Materials and methods

### 2.1. Experimental design and field data collection

A field (33.727239 N, 83.299097 W) was planted with cotton seed at the Iron Horse Farm of the University of Georgia in Watkinsville, Georgia, USA on 13 June 2016. The field contained 128 plots (16 rows with 8 plots per row) of length 3.05 m, with 1.83 m alleys between consecutive plots in a row, and row-spacing of 1.52 m (see Figure S1 in Supplementary Material). Three experimental genotypes (GA2011158, GA2009037, and GA2010074) and one commercial variety (Americot conventional) were used, each having 32 plot replicates. A completely randomized design was used to assign genotypes to individual plots. In each plot, 15 cotton seeds were manually planted at a spacing of 0.15 m. The first seed was planted 0.15 m away from the plot starting point, and hence a total of 15 seeds occupied 2.4 m out of 3.05 m in each plot. Cotton fiber was handpicked and weighed for 96 randomly selected plots (24 per genotype) on 4 November 2016 (which was 144 days after planting, DAP 144).

**Figure 1:**
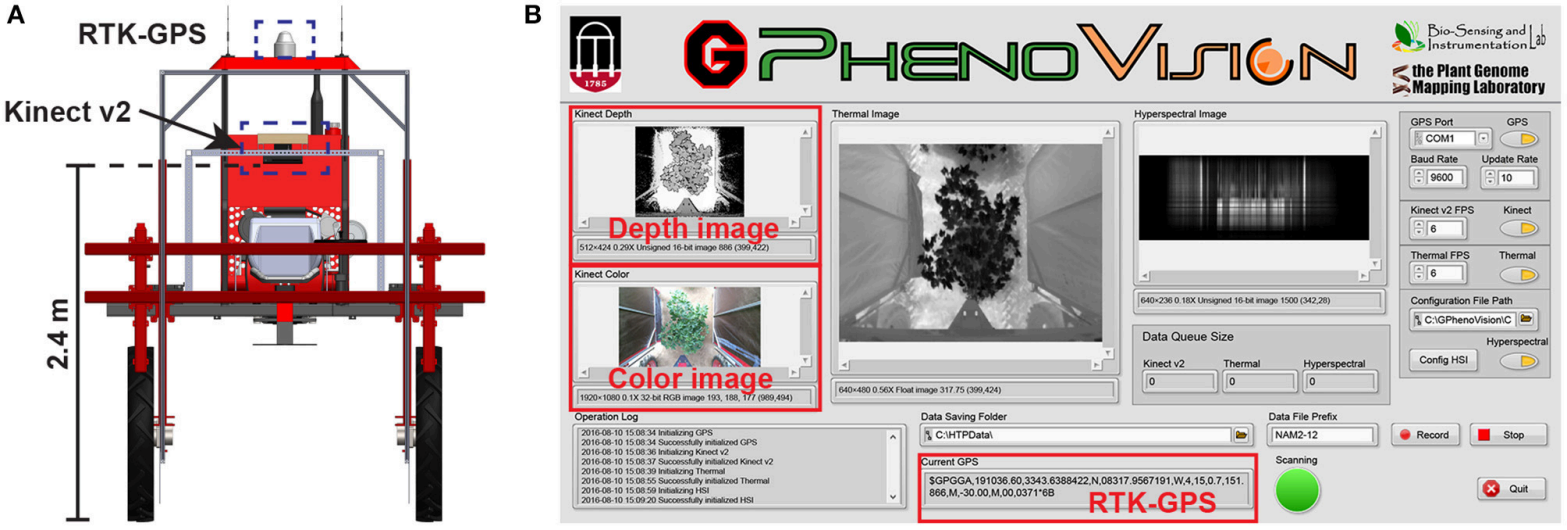
Field based phenotyping system used in this study: **(A)** Diagram of the camera and RTK-GPS configuration and **(B)** the front panel of data acquisition software developed using LabVIEW.

A field-based phenotyping system, GPhenoVision, was developed (Jiang et al., 2017) and used to scan the experimental field during midday (around 1,200–1,330 h) on 8 days in 2016 including 28 July (DAP 45), 4 August (DAP 52), 19 August (DAP 67), 26 August (DAP 74), 9 September (DAP 88), 16 September (DAP 95), 23 September (DAP 102), and 30 September (DAP 109). The scanning period covered two growth stages: canopy development (DAP 45–74), and flowering and boll development (DAP 74 to DAP 109). During the scanning period, the Kinect v2 camera of the GPhenoVision system was consistently used at 2.4 m above ground level (Figure 1A). The system ran in a continuous scanning mode at a constant speed of 1 m/s, but the operator manually controlled the data acquisition software to start/stop saving images at the beginning/end of each row to save storage space (Figure 1B).

### 2.2. Image processing pipeline

#### 2.2.1. Reconstruction of colorized point cloud

**Figure 2:**
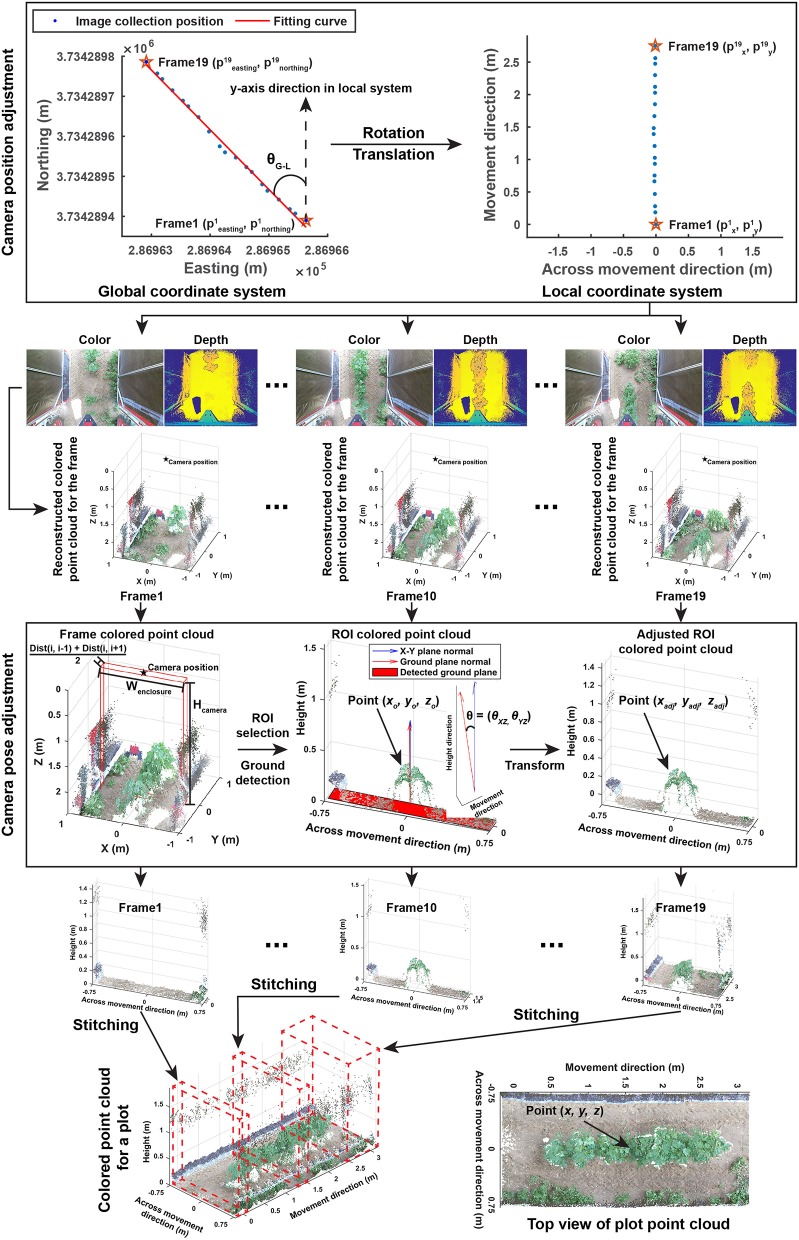
Processing pipeline of reconstructing colorized point clouds for individual plots using color and depth images and GPS collected by the GPhenoVision system.

The entire image processing pipeline included three sections: point cloud reconstruction, cotton canopy segmentation, and trait extraction. The aim of the point cloud reconstruction section was to reconstruct colorized point clouds for individual plots using collected depth and color images with their corresponding GPS information (Figure 2). This section contained four steps:

##### 2.2.1.1. Step 1.1: data grouping

All acquired GPS records were firstly converted from the geographic coordinate system to the universal transverse mercator (UTM) coordinate system. Altitude measures were used as the height of image acquisition. Collected depth and color images were segregated into individual plots based on their GPS coordinates, and the following processes were executed within each plot.

##### 2.2.1.2. Step 1.2: camera position adjustment

Each plot used both global and local coordinate systems. The global (UTM and altitude) system indicated exact positions of image acquisition (also image center), whereas the local system represented image pixel coordinates in which the y-axis was aligned with the vehicle moving (row) direction and the x-axis was aligned with the direction that is perpendicular to the vehicle moving (across-row) direction. Global (UTM and altitude) coordinates were converted to local coordinates, so image acquisition positions were aligned with image pixel coordinates. First, global coordinates were rotated to be aligned with y-axis in the local system using Equation (1).

(1) $$\begin{array}{l} \left[\begin{array}{c} p_{x}^{i}\\ p_{y}^{i}\\ p_{z}^{i} \end{array}\right]=\left[\begin{array}{ccc} \cos\theta_{G-L} & \sin\theta_{G-L} & 0\\ -\sin\theta_{G-L} & \cos\theta_{G-L} & 0\\ 0 & 0 & 1 \end{array}\right]\left[\begin{array}{c} p_{easting}^{i}-p_{easting}^{1}\\ p_{northing}^{i}-p_{northing}^{1}\\ p_{altitude}^{i}-p_{altitude}^{1} \end{array}\right] \end{array}$$

where θ_*G*−*L*_ was the rotation angle (the angle between the fitting curve and north axis) in radians, $p_{x}^{i}$, $p_{y}^{i}$, $p_{z}^{i}$were the x, y, z coordinates of the acquisition position of the *i*th frame in the local system, and $p_{easting}^{i}$, $p_{northing}^{i}$, and $p_{altitude}^{i}$ were the UTM coordinates and altitude values, respectively, of the acquisition position of the *i*th frame in the global system. *p*^1^ was the starting (first) frame acquired in each plot.

After the coordinate system conversion, image acquisition positions were aligned with image pixel coordinates and the starting point of each plot became the origin point in the local system. Individual depth and color images were reconstructed to colorized point clouds using functions provided by the manufacturer's software development kit (SDK).

##### 2.2.1.3. Step 1.3: camera orientation adjustment

The Kinect v2 camera might be slightly tilted during data collection due to uneven terrain, and thus it was necessary to estimate camera orientation in each frame and to correct point cloud offset due to camera orientation changes. As individual frames would be eventually stitched, only a part of the frame, the region of interest (ROI), needed to be processed. To save processing time, camera orientation estimation and adjustment were performed on the ROI of each point cloud. ROIs were selected based on image acquisition positions using Equation (2).

(2) $$\begin{array}{l} \left[\begin{array}{cc} ROI_{X}^{Lower} & ROI_{X}^{Upper}\\ ROI_{Y}^{Lower} & ROI_{Y}^{Upper}\\ ROI_{Z}^{Lower} & ROI_{Z}^{Upper} \end{array}\right]=\left[\begin{array}{cc} -\frac{W_{enclosure}}{2} & \frac{W_{enclosure}}{2}\\ -\frac{Dist\left(i,i-1\right)}{2} & \frac{Dist\left(i,i+1\right)}{2}\\ 0 & H_{camera} \end{array}\right] \end{array}$$

where $ROI_{\cdot }^{Lower}$ and $ROI_{\cdot }^{Upper}$ were the lower and upper limits on a specific axis of an ROI in the point cloud of a single frame, *W*_*enclosure*_ was the width of the enclosure (1.52 m in the present study), *Dist*(*i, j*) was the absolute distance difference in acquisition position between the *i*th and *j*th frames, and *H*_*camera*_ was the height of the camera above the ground level (2.4 m in the present study).

In each selected ROI, ground surface was detected using maximum likelihood estimation sample consensus (MLESAC) (Torr and Zisserman, 2000), and the normal of the detected ground surface was calculated. Angle differences in the normal between the detected and ideal (X-Y plane) ground surfaces were calculated, and points in the ROI were rotated based on the angle differences with respect to the X-Z and Y-Z planes using Equation (3).

(3) $$\begin{array}{l} \left[\begin{array}{c} x_{adj}\\ y_{adj}\\ z_{adj} \end{array}\right]=\left[\begin{array}{ccc} cos(\theta_{xz}) & 0 & sin(\theta_{xz})\\ 0 & 1 & 0\\ -sin(\theta_{xz}) & 0 & cos(\theta_{xz}) \end{array}\right]\left[\begin{array}{ccc} 1 & 0 & 0\\ 0 & cos(\theta_{yz}) & sin(\theta_{yz})\\ 0 & -sin(\theta_{yz}) & cos(\theta_{yz}) \end{array}\right]\left[\begin{array}{c} x_{o}\\ y_{o}\\ z_{o} \end{array}\right] \end{array}$$

where θ_*xz*_ and θ_*yz*_ were the angles between the detected and ideal ground surfaces with respect to the X-Z and Y-Z planes, and *x*_*adj*_, *y*_*adj*_, and *z*_*adj*_ were the adjusted coordinates of the original points (*x*_*o*_, *y*_*o*_, and *z*_*o*_) in the ROI.

If vegetation covered the entire ground surface and resulted in a failure of ground detection, the ground normal of the ROIs was assumed to be the same as the X-Y plane normal (${\vec{n}}_{X-Y}=\left[0,0,1\right]$). As a consequence, there was no adjustment to account for effects caused by camera orientation changes.

##### 2.2.1.4. Step 1.4: stitching of selected ROIs

The final step in the reconstruction section was to stitch all of the selected ROIs into an integrated colorized point cloud for individual plots. Based on camera positions, the transformation of points in ROIs to the local coordinate system would naturally result in stitching of ROIs and produce the colorized point cloud for a plot. The coordinate transformation was conducted using Equation (4).

(4) $$\begin{array}{l} \left[\begin{array}{c} x\\ y\\ z \end{array}\right]=\left[\begin{array}{c} x_{adj}\\ y_{adj}\\ z_{adj} \end{array}\right]+\left[\begin{array}{c} p_{x}^{i}-p_{x}^{1}\\ p_{y}^{i}-p_{y}^{1}\\ p_{z}^{i}-p_{z}^{1} \end{array}\right] \end{array}$$

where *x, y*, and *z* were the coordinates of points in the final colorized point cloud of a plot, and $p_{x}^{i}$, $p_{y}^{i}$, and $p_{z}^{i}$ were the coordinates of the acquisition position of the *i*th frame in the local coordinate system.

#### 2.2.2. Segmentation of cotton plant canopies

**Figure 3:**
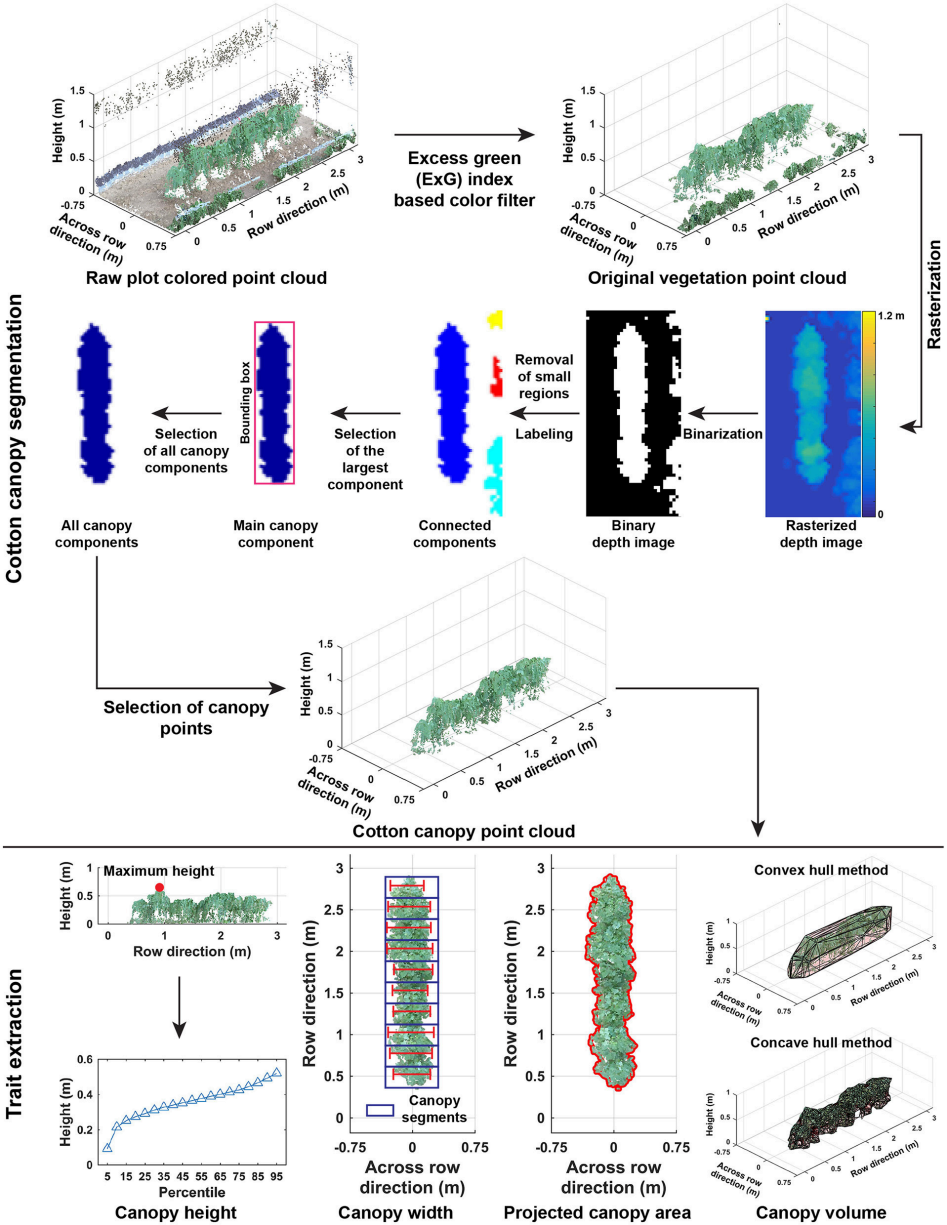
Processing pipeline of segmenting cotton canopy from the reconstructed point clouds and of extracting morphological traits from the canopy point clouds.

Point clouds of cotton plots could contain irrelevant objects such as system frames, ground surface, and weeds. Therefore, it was necessary to segment the cotton plant canopy from irrelevant objects (Cotton canopy segmentation in Figure 3). The segmentation of cotton canopies involved two steps:

##### 2.2.2.1. Step 2.1: vegetation segmentation

Unlike point cloud data collected by conventional instruments such as LiDAR, point cloud data in the present study also contained color information (RGB values) for each point. A color filter was used to segment vegetation from background objects. According to previous studies (Hamuda et al., 2016), excess green (ExG) index was an effective indicator to obtain vegetation objects in an image, and thus ExG values were calculated for each point of a plot. A preliminary test was performed on a small subset of the collected images, showing that a threshold of 0.15 provided adequate separation between vegetation and background.

##### 2.2.2.2. Step 2.2: cotton canopy segmentation

Color filtering was able to remove most irrelevant objects but not weeds, because plants and weeds are both vegetation and thus hard to differentiate based on color information. In this step, point clouds were rasterized to depth images, so pixels of cotton canopies were differentiated from those of weeds in the depth images by using 2D computer vision algorithms with spatial information including height (depth), area, and position. Identified canopy pixels were back-projected to 3D space to select points representing cotton canopies in point clouds. Point cloud data (x, y, and z coordinates) were rasterized to depth images using Equation (5).

(5) $$\begin{array}{l} I_{D}(i,j)=\max(z_{k}),\\ \;\forall k\in \{k|x_{Lower}+S_{g}\times (i-1)⩽x_{k}⩽x_{Lower}+S_{g}\times i\}\\ \;\cap \{k|y_{Lower}+S_{g}\times (j-1)⩽y_{k}⩽y_{Lower}+S_{g}\times j\}\\ \;i=1,2,\ldots ,\lceil \frac{Range(x)}{S_{g}}\rceil \\ \;j=1,2,\ldots ,\lceil \frac{Range(y)}{S_{g}}\rceil \end{array}$$

where *I*_*D*_ represented a rasterized depth image, and *i* and *j* were pixel indices in *I*_*D*_. *x*_*Lower*_ and *y*_*Lower*_ were the lower limits of vegetation point clouds, and *S*_*g*_ was the grid size (0.05 m in the present study). *x, y*, and *z* were coordinates of vegetation points, and *k* was the index of a point in vegetation point clouds. *Range*(·) was a function to calculate the maximum length along a particular axis in vegetation point clouds, and $\left\lceil \frac{Range\left(\cdot \right)}{S_{g}}\right\rceil$ was the total number of grid cells along a particular axis.

Most in-row weeds were short and small, and removed using height and area information. Depth images were binarized by the 30th percentile of depth value of all pixels in order to exclude those representing short weeds. Connected components (CCs) were labeled in binary depth images, and small CCs representing weeds were removed using Equation (6). Thresholds of depth and area were empirical values based on observations of height and area of weeds in the present study.

(6) $$\begin{array}{l} \begin{array}{cc} CC_{i}^{included}=\left\{ \begin{array}{cc} 1,\; & Area\left(CC_{i}\right)>=Th_{Area}\\ 0,\; & \text{otherwise} \end{array}\right., & i=1,2,\ldots ,n \end{array} \end{array}$$

where *CC*^*included*^ is a flag for a connected component, and “1” or “0” indicated inclusion/exclusion of the connected component in the binary depth image. *Area*(·) was a function to count the number of pixels in a connected component, and *Th*_*Area*_ was the threshold (15 pixels in the present study) for including a CC.

Weeds between plots were tall and large, and removed using position information. The CC with the largest area (pixel counts) was selected as the main canopy in a plot, and its bounding box was calculated. Based on their positions relative to the main canopy, remaining CCs were classified as weed or cotton canopy using Equation (7).

(7) $$\begin{array}{l} \begin{array}{cc} CC_{i}^{class}=\left\{ \begin{array}{cc} 1,\; & Dist\left(CC_{i},CC_{m}\right)⩽W_{CC_{m}^{b}}\\ 0,\; & \text{otherwise} \end{array}\right., & \forall i\in \left\{i|CC_{i}^{included}=1\right\} \end{array} \end{array}$$

where *CC*^*class*^ is the class marker for a CC with “1” for cotton canopy and “0” for weeds. *Dist* was a function to calculate the distance between the center position of the *i*th CC and main canopy CC (*CC*_*m*_), and $W_{CC_{m}^{b}}$ was the width of the bounding box for main canopy CC.

According to the rasterization process, a pixel in depth images represented a grid cell in vegetation point clouds. Based on Equation (5), each pixel in the identified canopy CCs was back-projected to a grid cell, and vegetation points in that cell needed to be included in canopy point clouds. Vegetation points were selected using Equation (8) to form canopy point clouds that were used for trait extraction in each plot.

(8) $$\begin{array}{l} \begin{array}{cc} PtCloud_{canopy}=\left\{\forall pt|pt\in CC_{i}\right\}, & i=\left\{j|CC_{j}^{class}=1\right\} \end{array} \end{array}$$

where *PtCloud*_*canopy*_ represented point clouds of cotton canopy, *pt* was a point in vegetation point clouds, *CC*_*i*_ was the *i*th identified canopy CC, and $CC_{j}^{class}$ was the class marker of the *j*th CC in a depth image.

#### 2.2.3. Extraction of morphological traits

Morphological traits were extracted from point clouds of cotton plant canopies (Trait extraction in Figure 3). Trait extraction included two parts: extraction of static traits from multiple dimensions (one- and multi-dimensional traits) and calculation of dynamic traits (growth rates).

##### 2.2.3.1. Step 3.1: extraction of one-dimensional traits

One-dimensional traits contained canopy height (maximum and mean), cumulative height profile, and width (maximum and mean) at the plot level. The maximum and mean canopy heights were defined as the tallest and average height values of all canopy points. Cumulative height profile was the combination of canopy height from the 5th percentile to 95th percentile with an interval of 5 %. For canopy width, the point cloud of a cotton canopy was segregated into ten segments along the row direction, and the maximum length across the row direction was calculated in each segment. Maximum and mean widths were the maximum and average values of widths in the ten segments.

##### 2.2.3.2. Step 3.2: extraction of multi-dimensional traits

Multi-dimensional traits contained projected canopy area and canopy volume. All canopy points were projected onto the X-Y plane, and the boundary of the projected shape was identified to calculate the projected canopy area. Convex and concave hulls were detected on canopy point clouds, and canopy volume was estimated using the detected convex and concave hulls.

##### 2.2.3.3. Step 3.3: calculation of growth rate

In addition to static traits on a specific date, growth rates (dynamic changes) could provide information about growth behavior for cotton plants. Growth rate was defined and calculated between every two consecutive data collection dates using Equation (9).

(9) $$\begin{array}{l} G_{T,P}=\frac{T_{d_{last}}-T_{d_{first}}}{d_{last}-d_{first}} \end{array}$$

where *G* was the growth rate of a trait (*T*) during a period (*P*). *d*_*first*_ and *d*_*last*_ were the first and last days after planting in the period (*P*).

### 2.3. Performance evaluation

#### 2.3.1. Accuracy of canopy segmentation

Cotton canopy segmentation strongly affected the accuracy of trait extraction. In particular, weed removal would significantly influence values of extracted morphological traits. Ground truth segmentation results of depth images were manually generated, and accuracy, false positive rate (FPR; weed pixels identified as canopy), and false negative rate (FNR; canopy pixels identified as weeds) were calculated for each plot. Based on these three values, segmentation results were evaluated and classified into three categories: clean canopy (accuracy ⩾ 95%), under-removal of weed (FPR ⩾ 10%), and over-removal of canopy (FNR ⩾ 10%). The percentages of the three categories were used as indicators to evaluate the performance of canopy segmentation.

#### 2.3.2. Accuracy of sensor measurements

It was important to evaluate the accuracy of depth measurement because depth information was not only used for height measurement but also calculation of x and y coordinates of individual points, thereby affecting the overall accuracy of obtained point clouds. As height was directly derived from depth measurement, maximum canopy height was measured for 32 field plots that were randomly selected on each of four scanning dates (DAP 45, 52, 74, and 88). Therefore, a total of 128 data points were obtained for evaluating the accuracy of height measurement.

In addition, due to difficulties in field measurement, potted and artificial plants were used to assess accuracies of measuring other traits. A total of 8 potted plants were used for validating measurements of width, length, and volume, and an artificial plant was used for projected leaf/convex area (see Figure S2). For the potted plants, canopy width (cross-movement direction) and length (movement direction) were measured using a ruler, whereas volume was measured using a protocol for tree volume estimation (Coder, 2000) (see Figure S3). Following the protocol, a plant was virtually segregated into layers with a height interval of 5 cm. Diameters were measured for the middle of individual layers, from which volume could be estimated using a cylinder model, and the plant volume was the summation of all layer volumes. For the artificial plant, a total of 8 layouts were configured to form different plant leaf/convex areas (see Figure S4). In each layout, all leaves were laid on the base surface and imaged with a size marker by a digital single-lens reflex (DSLR) camera (A10, Fujifilm Holdings Corporation, Tokyo, Japan) so that projected leaf/convex area could be accurately measured using image processing. After taking color images, leaves were vertically lifted at various heights to form a 3D layout simulating real plants. It was noteworthy that real plants had many more leaves than the artificial plant, resulting in a denser canopy, and thus projected convex area would be closer to projected canopy area in real situations. In total, 8 data points were used for validating sensor measurements of canopy width, length, projected area, and volume.

Simple linear regression analyses were performed between sensor and manual measurements for all traits. Adjusted coefficient of determination (Adjusted *R*^2^) and root mean squared error (RMSE) were used as indicators for performance assessment.

#### 2.3.3. Efficiency of image processing

In addition to accuracy performance, the efficiency of the proposed algorithm was tested on a workstation computer that used an Intel i7-4770 CPU with 16 GB of RAM on a Windows 10 operating system. Processing time of point cloud reconstruction, cotton canopy segmentation, and trait extraction was recorded during the processing of all plot data collected on all eight data collection dates. For point cloud reconstruction, simple linear regression analysis was conducted between the number of frames in each plot and the reconstruction time. For cotton canopy segmentation and trait extraction, the percentage of processing time for each key step was calculated.

### 2.4. Statistical analyses between fiber yield and extracted traits

Extracted traits were grouped into two categories: univariate and multivariate traits. For univariate traits, simple linear regression analyses were conducted between the traits and fiber yield, whereas for multivariate traits, multiple linear regression analyses were conducted between the traits and fiber yield. The adjusted *R*^2^ and RMSE were used to assess the potential of the usefulness of extracted traits for prediction of cotton fiber yield. As regression models established using the maximum and mean canopy height were nested to those using cumulative height profile, *F*-tests were conducted to rigorously test the statistical significance of model differences. All regression and *F*-test analyses were performed in R software (R Core Team, 2016).

## 3. Results

### 3.1. Performance of segmentation of cotton canopy

**Figure 4:**
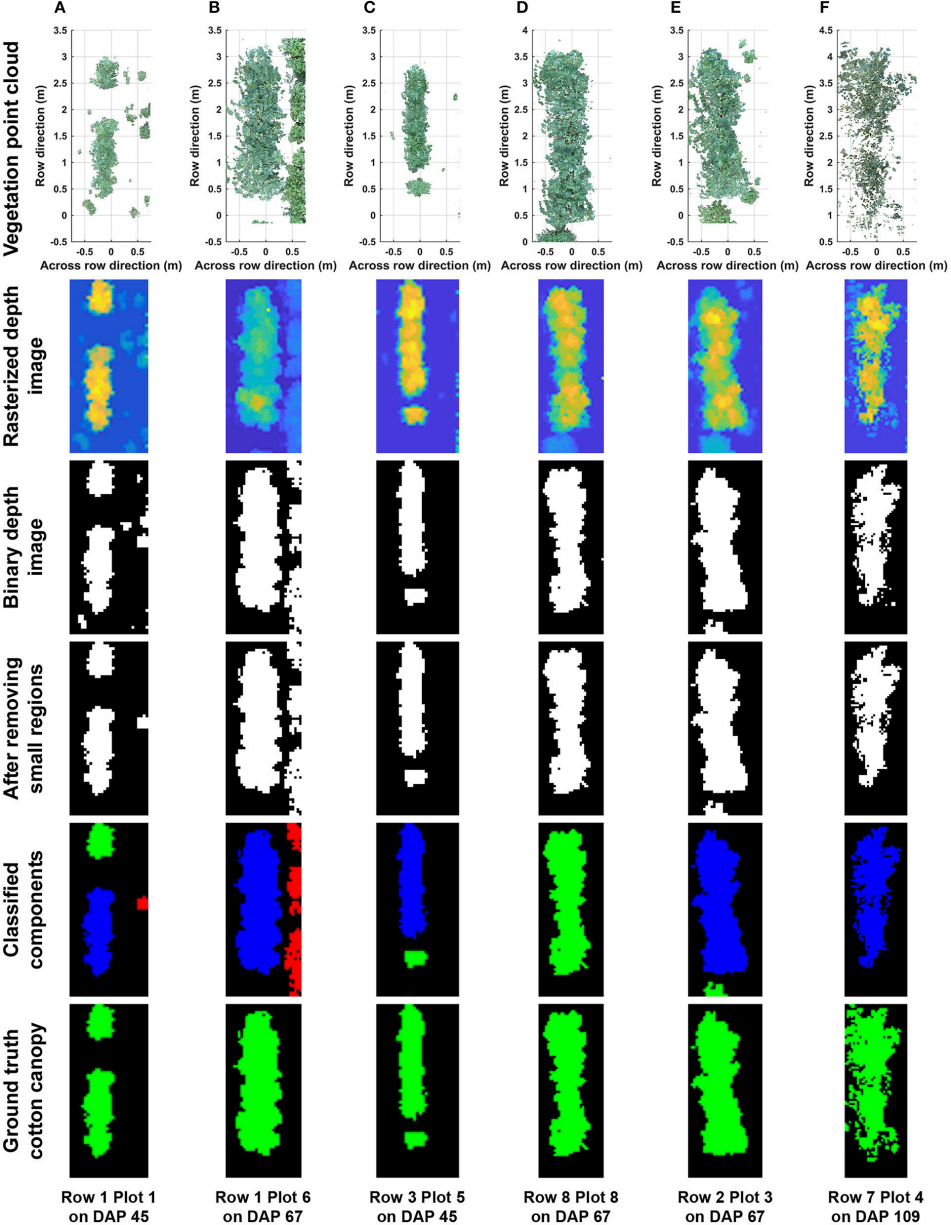
Representative results of cotton canopy segmentation: successful segmentation under **(A)** poorly germinated plot with weeds, **(B)** well germinated plot with weeds, **(C)** plot with a segregated plant, and **(D)** well germinated plot with connected weeds; **(E)** failure of weed removal; and **(F)** over-removal of cotton plant canopy. In classified connected component images, blue, green, and red colors indicated main canopy, cotton canopy, and weeds, respectively. Ground truth images were manually generated for including cotton canopy.

Overall, the cotton canopy segmentation achieved an accuracy of 98.6% on all collected data, which indicates that the scanning system provided accurate canopy point clouds for trait extraction throughout the growing season (Table 1). The proposed algorithms precisely segmented cotton canopy under various plot conditions (Figures 4A–D). In-row weeds were usually shorter and smaller than cotton plants, so they were mostly removed by height and area filters. In contrast, weeds between rows were generally large and tall, but they could be removed using position information because they were located between plots and away from the main canopy in each plot. Therefore, the method could provide accurate segmentation of cotton plant canopy. However, the weed under-removal rate increased noticeably on DAP 67 and DAP 74. This was because no weeding activity was arranged during that period, and many weeds grew substantially and became comparable with cotton plants in height and size; as a consequence, cotton plants became hard to differentiate from weeds (Figure 4E). In late growth stages (e.g., flowering and boll development stage), cotton plant leaves started to shrink and fall down, resulting in a reduction of canopy size and overlap. Large canopies were segregated into small leaf areas, especially along the outer part of canopies. These small leaf areas were largely filtered out by the area filter, leading to over-removal of canopy (canopy pixels identified as weeds) (Figure 4F).

**Table 1:** Performance of the segmentation of cotton canopy.

**Date**	**Clean canopy (%)**	**Weed under-removal (%)**	**Weed over-removal (%)**
28 July 2016 (DAP 45)	99.2	0.8	0
4 August 2016 (DAP 52)	99.2	0.8	0
19 August 2016 (DAP 67)	95.2	4.8	0
26 August 2016 (DAP 74)	97.6	1.6	0.8
9 September 2016 (DAP 88)	99.2	0.8	0
16 September 2016 (DAP 95)	100	0	0
23 September 2016 (DAP 102)	100	0	0
30 September 2016 (DAP 109)	98.4	0	1.6
All dates	98.6	1.1	0.3

### 3.2. Representative colorized point clouds

**Figure 5:**
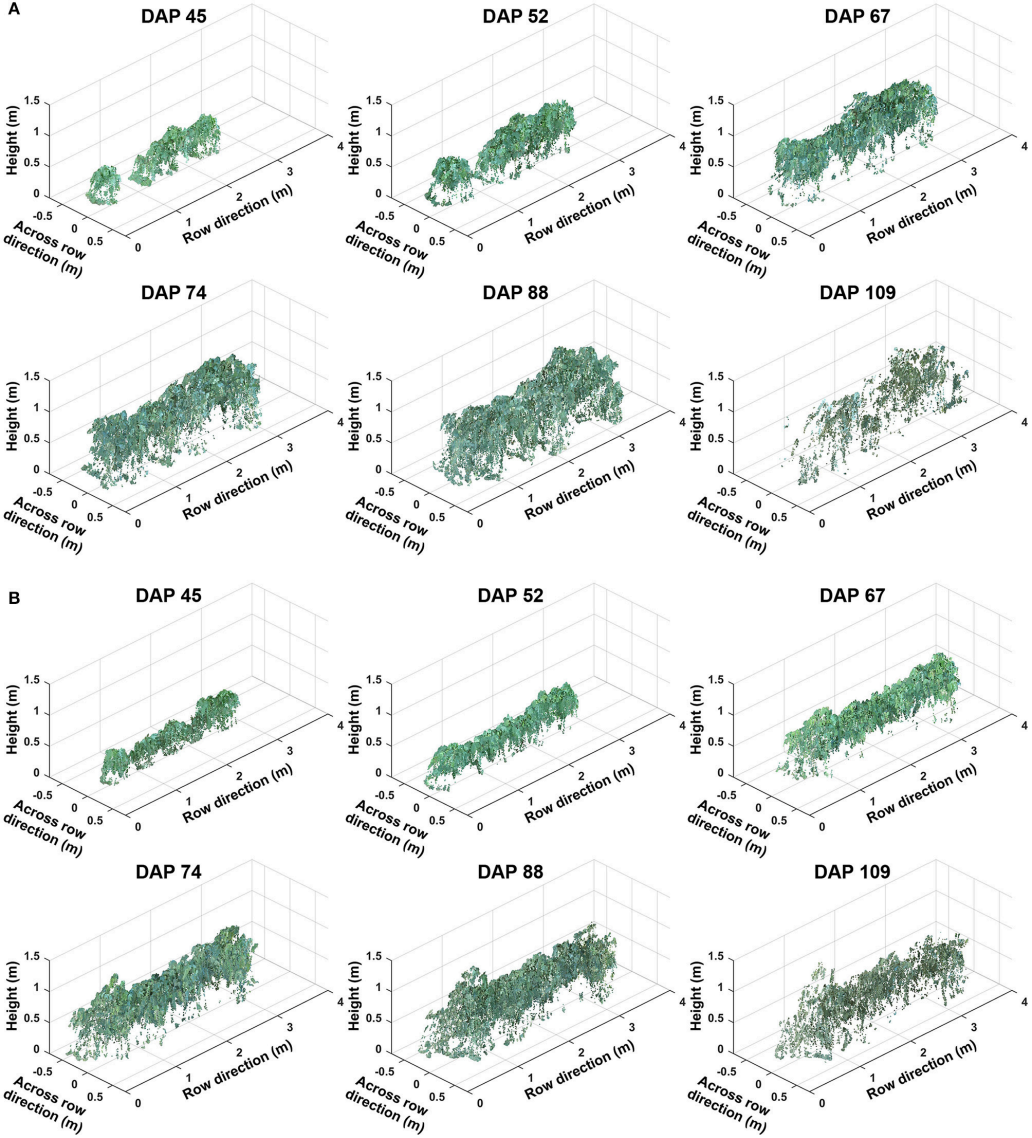
Representative point clouds of the same plot during the growing season: **(A)** A plot with poor germination rate (less than 50%) and **(B)** a plot with good germination rate (greater than 75%). Collected data covered two growth stages: (1) canopy development from days after planting (DAP) 45 to 74 and (2) flowering and boll development from DAP 74 to DAP 109.

Representative colorized point clouds of cotton canopies were generated and demonstrated at the plot level over the growing season (Figure 5). Poorly germinated plots contained some empty areas (Figure 5A), which tended to be at least partly filled by neighboring plants after the canopy development stage (DAP 74). In well germinated plots, seeds generally sprouted around the same time and seedlings grew at comparable speeds, maintaining similar canopy height along a plot. Even in cases with some variation of germination date (thus development speed), canopy height tended to reach a similar level in a plot, avoiding the occurrence of extremely tall or short sections (Figure 5B). Well germinated plots showed canopy overlap at an earlier date (approximately DAP 52 or earlier) than poorly germinated ones and were relatively flat (or slightly arched) along the row direction, whereas in poorly germinated plots the canopy close to empty areas was short. This slightly arched shape of the canopy is due to “edge effect,” and plants can be either bigger or smaller at the plot edge than those in the center.

### 3.3. Accuracy of sensor measurement

**Figure 6:**
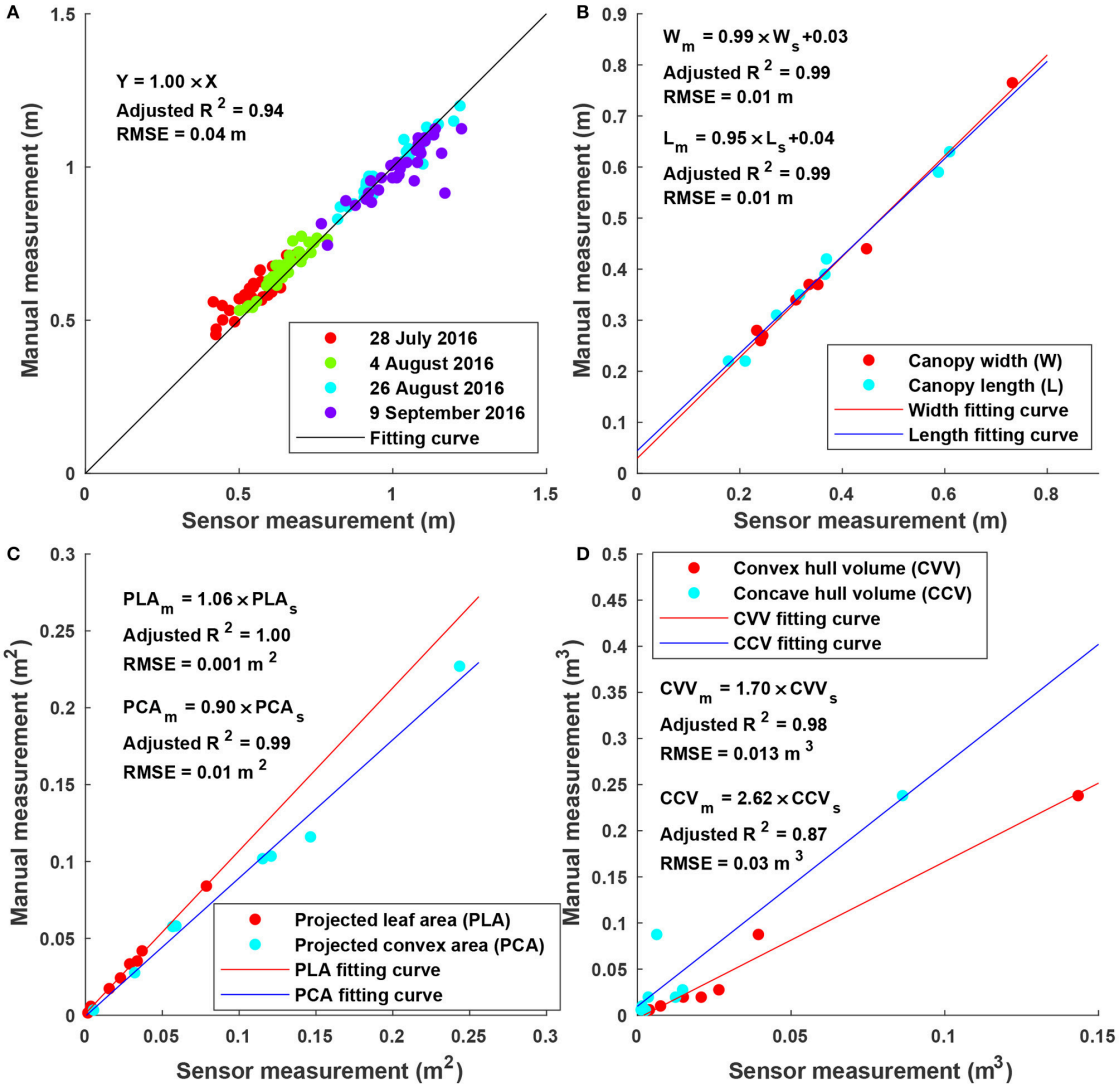
Linear regression results between sensor and manual measurements for height, width, length, projected leaf/convex area, and volume: **(A)** Maximum canopy height (*N* = 128) for individual plots on four dates; **(B)** canopy width and length (*N* = 8 for each) for potted plants; **(C)** projected leaf and convex area (*N* = 8 for each) for the artificial plant with different layouts; and **(D)** volume (*N* = 8) for potted plants.

Sensor measurements were strongly correlated (adjusted *R*^2^ > 0.87) with manual measurements for all extracted traits (Figure 6). In particular, height measurements were obtained from multiple days and the RMSE was 0.04 m, suggesting a high accuracy and repeatability of depth measurement (and therefore point clouds) in field conditions. Consequently, point clouds acquired by the Kinect v2 sensor could be used to accurately measure traits such as width, length, projected leaf/convex area. It should be noted that although correlation was strong (adjusted *R*^2^ > 0.87) between sensor and manual measurements for volume, the absolute values were significantly different (see the slope in regression equations for convex and concave hull volumes). Convex and concave hull volumes were smaller than reference measurements. In the present study, the Kinect v2 sensor acquired point clouds from the top view, and thus only canopy surface could be imaged, resulting in a lack of information from two sides (especially sections under the canopy). As a consequence, the volume of a plant (or a plot) estimated by convex and concave hulls was a portion of the ground truth value. Since the canopy of most cotton plants was roughly a cone, the sensed portion could generally represent the entire plant. This was also supported by the high correlation between sensor and reference measurements. In addition, convex hull volume showed better correlation with reference measurement than concave hull volume, because both convex hull and reference measurements included volume of empty space among branches of a plant. Nonetheless, as both convex and concave hull demonstrated strong correlation, they could be used as indicators for growth pattern analyses and/or yield prediction.

### 3.4. Efficiency of the proposed approach

**Figure 7:**
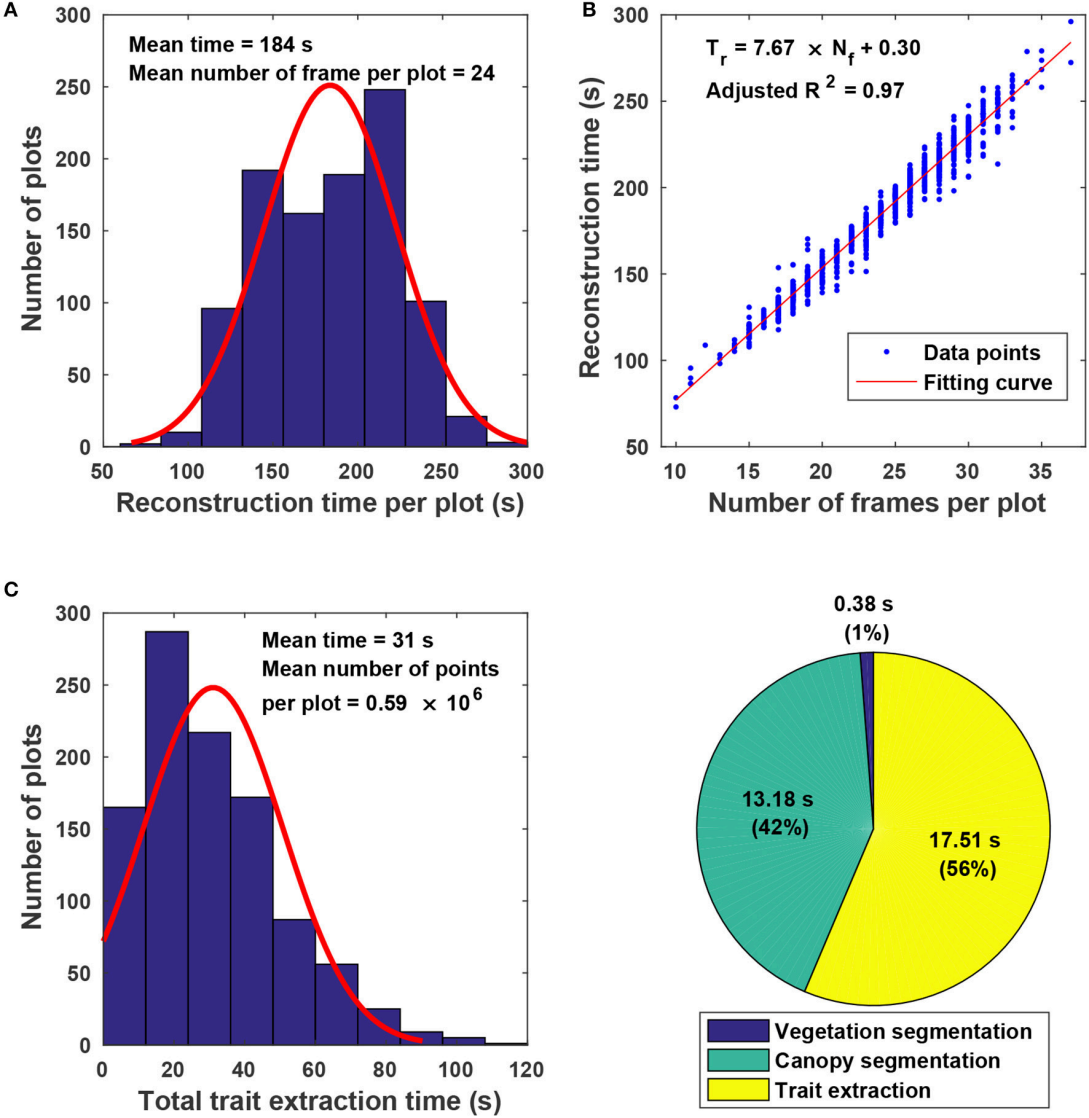
Algorithm efficiency of point cloud stitching and trait extraction: **(A)** Histogram of reconstruction time for individual plots; **(B)** relationship between the number of images collected in each plot and the reconstruction time of stitching; **(C)** histogram of the total trait extraction time for individual plots; and **(D)** percentages of processing time for various steps in canopy segmentation and trait extraction.

The proposed approach used on average 215 s for processing a plot, including 184 s for point cloud reconstruction and 31 s for canopy segmentation and trait extraction (Figure 7). Variations in reconstruction time primarily came from the different number of frames acquired for a plot (Figure 7A). Although the GPhenoVision system was set at a constant speed, the actual system speed would vary due to different terrain conditions (dry vs. wet) and scanning status slower at the two ends of a row than in the middle). Consequently, the number of frames could be different in various plots on different dates. Generally, the reconstruction time increased linearly with the number of frames acquired in a plot (Figure 7B). However, for plots with equal number of frames, variations in processing time were mainly due to different number of points in frames. For instance, point clouds of poorly germinated plots contained more points of ground than those of well germinated plots, resulting in variation in ground surface detection and thus in reconstruction time.

For canopy segmentation and trait extraction, efficiency variations occurred due to different numbers of points in point cloud data (Figure 7C). The number of points in a point cloud was determined by the canopy size, which was affected by both germination condition and growth stage. Plant canopies were larger in well germinated plots or plots in the canopy development stage than those in poorly germinated plots or plots in the flowering and boll development stage. Thus, the number of canopy points varied from plot to plot and stage to stage, resulting in processing time variations. Among the three operations, canopy segmentation and trait extraction were more time consuming than vegetation segmentation (Figure 7D). Vegetation segmentation was based on color filtering, in which all points were processed simultaneously. In contrast, canopy segmentation and trait extraction processed information point by point, thus using a significantly longer time than vegetation segmentation.

### 3.5. Growth trend of cotton plants

#### 3.5.1. Static traits

**Figure 8:**
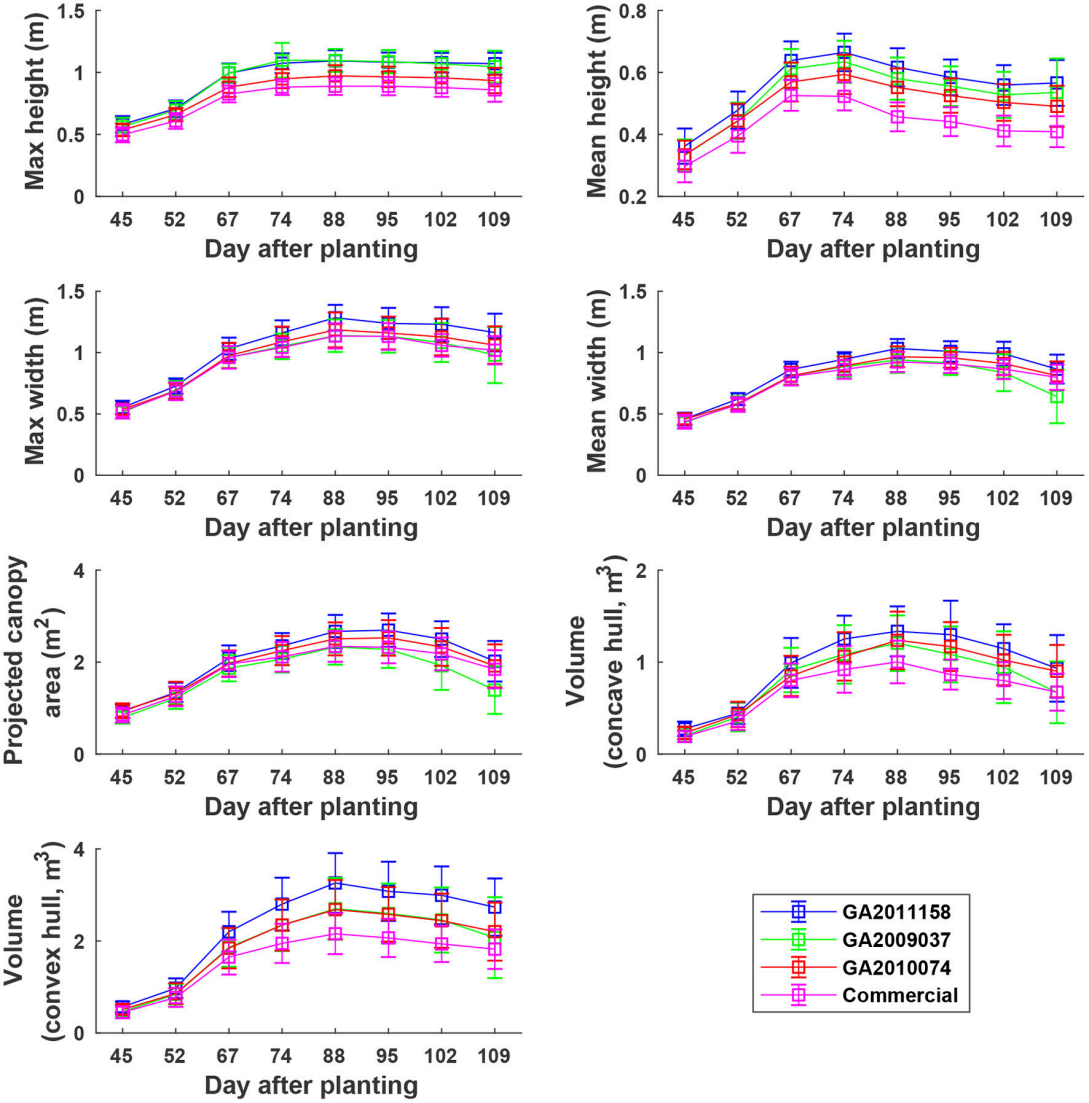
Extracted static traits of individual plots in ENGR field during the growing season. Extracted traits covered two growth stages: (1) canopy development from DAP 45 to DAP 74 and (2) flowering and boll development from DAP 74 to DAP 109.

Overall, plants elongated substantially from DAP 45 to DAP 74 (canopy development stage) and expanded considerably from DAP 45 to DAP 88 (canopy development stage and early flowering stage) (Figure 8). The values of canopy morphological traits gradually decreased during the rest of the growing season (flowering and boll development stages). Among the genotypes, the Americot conventional showed the shortest and smallest canopy, compared with the other three experimental genotypes. Genotype GA2009037 was the tallest genotype but with the least width, indicating a tall and narrow canopy (statistical comparison results are provided in Tables S1–S7).

**Figure 9:**
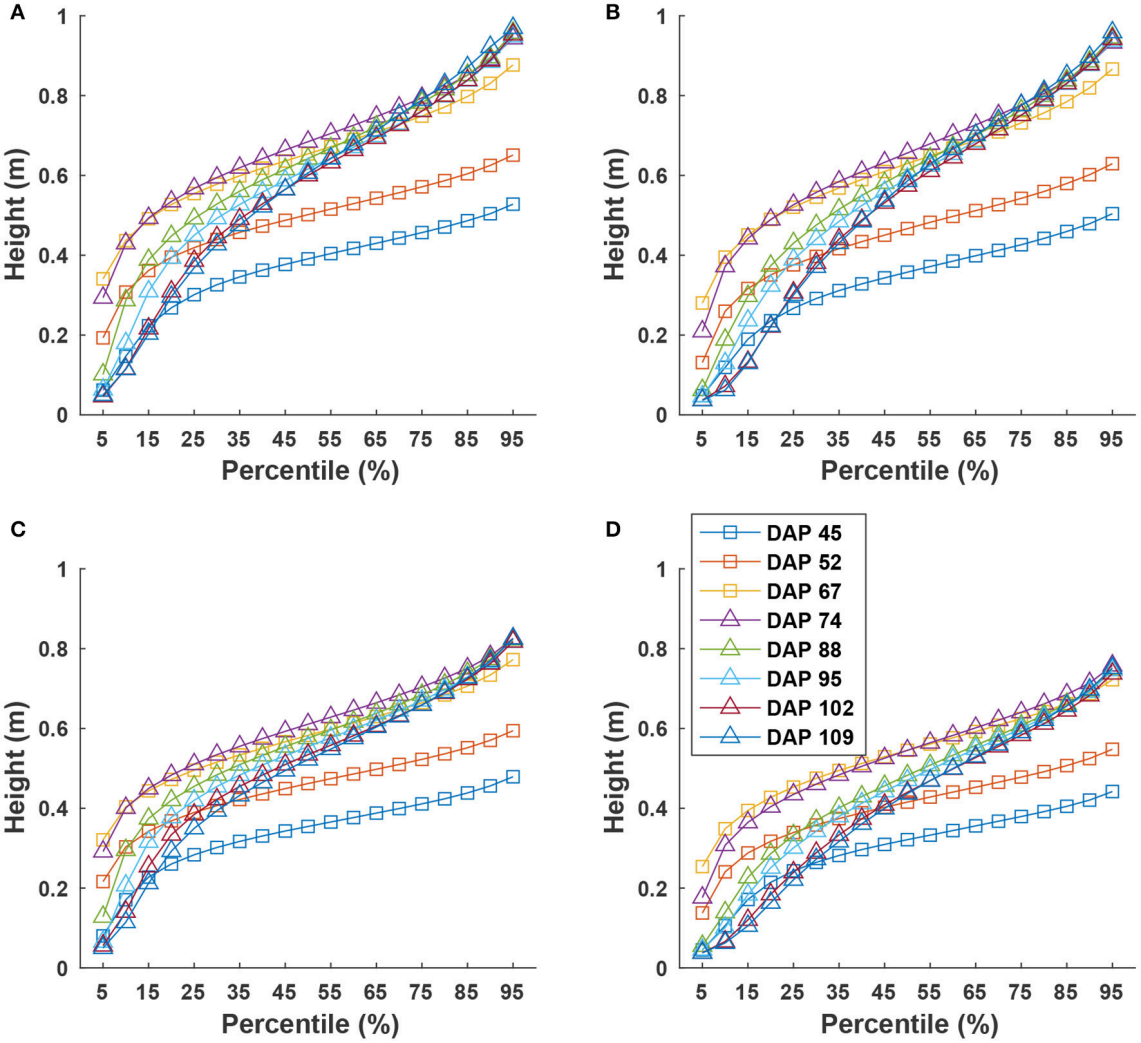
Extracted canopy height at various percentiles during the growing season: **(A–D)** were for GA2011158, GA2009037, GA2010074, and commercial variety. Extracted traits covered two growth stages: (1) canopy development from DAP 45 to DAP 74 and (2) flowering and boll development from DAP 74 to DAP 109.

Cumulative height profiles of all four genotypes showed logarithmic growth during the canopy development stage (DAP 45 to DAP 74) and linear growth during the flowering and boll development stage (DAP 74 to DAP 109) (Figure 9). In the canopy development stage, plant leaves increase in transpiration capability and maintain a solid shape to receive more sunlight for photosynthesis. Therefore, at the canopy development stage, upper leaves occluded lower ones, resulting in less variation in canopy height after the 25th percentile. The large difference between the 5th and 25th percentiles was due to un-occluded leaves on expanded branches at lower positions along the canopy border. When transitioning into flowering and boll development stage, plant leaves receive fewer nutrients (due to nutrient demands of maturing fruit) and start to shrink. Previously occluded leaves could be captured, and thus canopy height was represented by leaves at a wider range of positions, resulting in an increase of height difference at various percentiles. This information might be useful for predicting the change of growth stages for cotton plants.

#### 3.5.2. Dynamic traits

**Figure 10:**
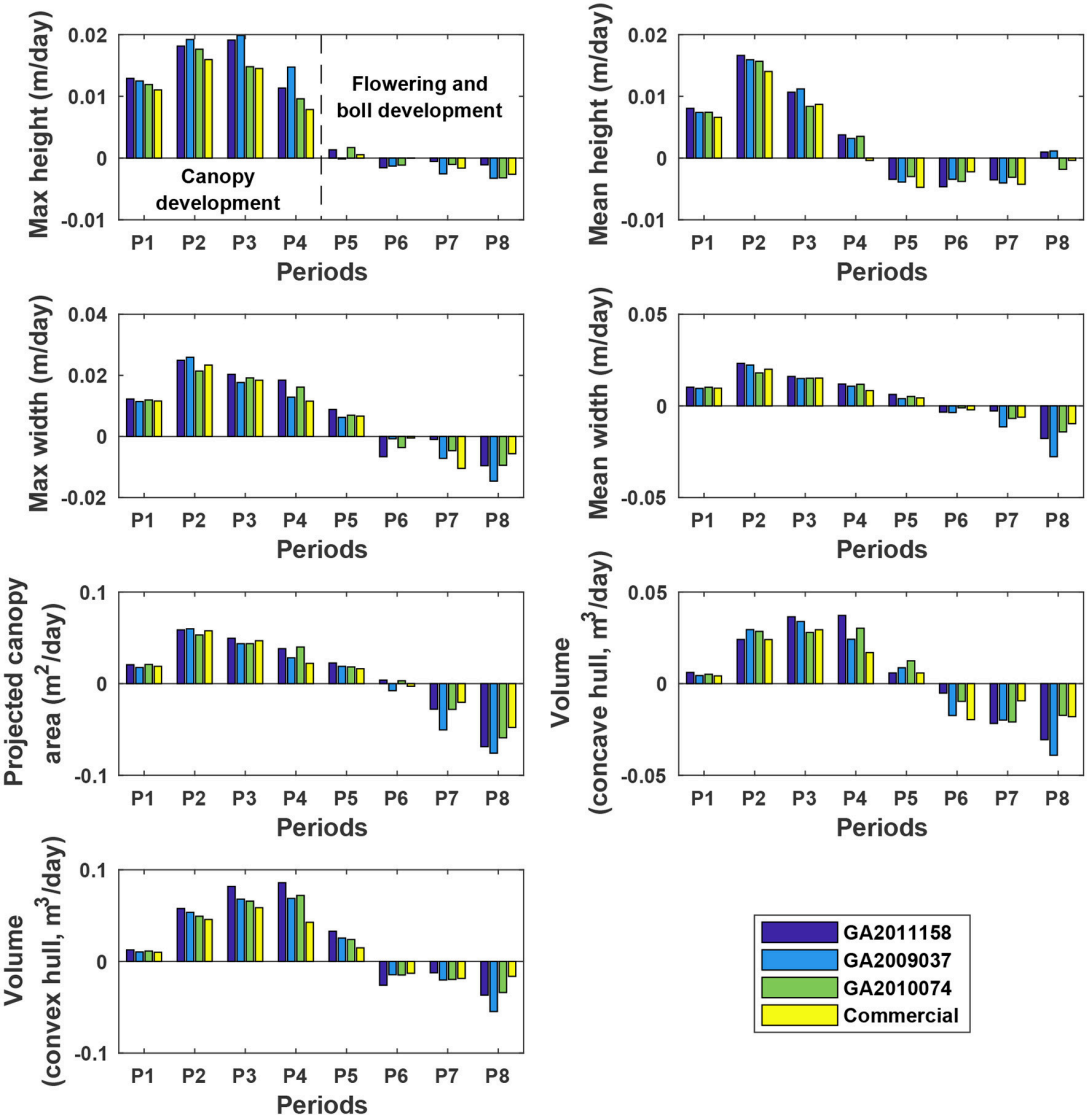
Extracted growth rates (dynamic traits) of individual plots in ENGR field during the growing season. P1 was the period from the day of planting to DAP 45, and P2–P8 were the periods between two consecutive data collection dates. Extracted traits covered two growth stages: (1) canopy development from P1 to P4 (DAP 67 to DAP 74) and (2) flowering and boll development from P4 to P8 (DAP 102 to DAP 109).

The four genotypes showed different patterns of canopy development in different growth stages (Figure 10). Generally, growth rates of all four genotypes approached zero in P5 (DAP 74 to DAP 88, which was the first data collection period in the flower and boll development stage) for maximum and mean canopy height, and in P6 (DAP 88 to DAP 95, which was the second data collection period in the flower and boll development stage) for the other traits, because the four genotypes were short season cottons and had similar growth stage transitions. In addition, all genotypes stopped canopy elongation approximately a week earlier than canopy expansion. Although the overall growth trend was similar, there were differences in growth behavior among the four genotypes (statistical comparison results are provided in Tables S8–S14). The Americot conventional was generally the slowest in developing its canopy, whereas the three experimental genotypes grew rapidly in different dimensions. Genotype GA2011158 showed the fastest growth in all dimensions, resulting in large canopy height, width, projected area, and volume. Genotype GA2009037 primarily elongated rather than expanded, resulting in tall canopies with the least projection coverage, whereas Genotype GA2010074 mainly expanded rather than elongated, resulting in short canopies with large coverage and volume. Detection of such variations might permit identifying genes controlling cotton plant growth patterns and/or selecting genotypes suitable for different production or harvesting strategies.

### 3.6. Performance of yield prediction

**Figure 11:**
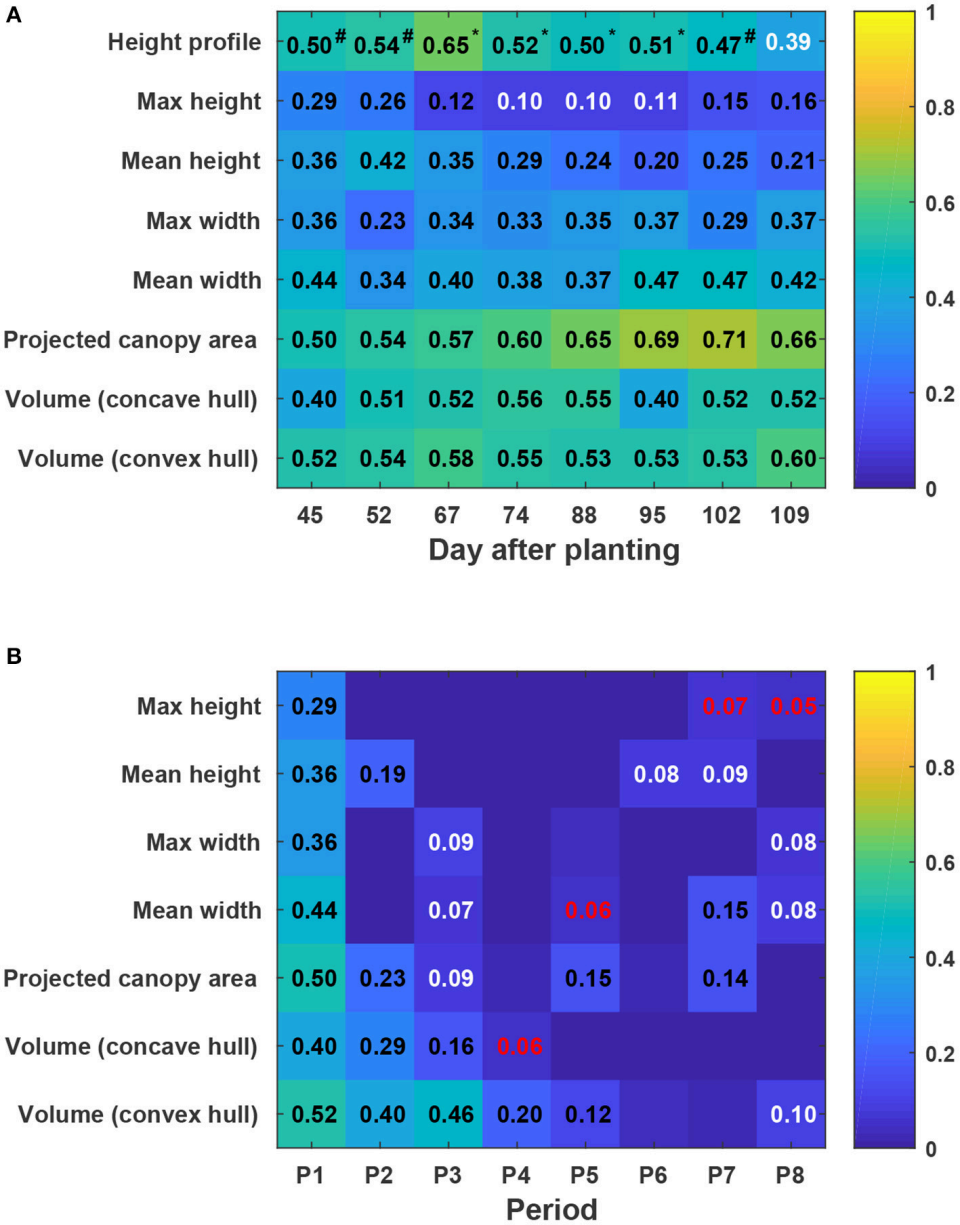
Coefficients of determination (*R*^2^) of linear regressions between extracted traits and cotton fiber yield: **(A)** Static traits and fiber yield and **(B)** dynamic traits and fiber yield. Black, white, and red colors indicated statistical significance of “<0.001,” “<0.01,” and “<0.05,” respectively. Insignificant *R*^2^ values were not shown in blocks. P1 was the period from the day of planting to DAP 45, and P2–P8 were the periods between two consecutive data collection dates. Superscripts of *R*^2^ values for height profile denoted the *F*-test results between regression models: a number sign (#) indicated a significant model improvement by using height profile rather than maximum height, and an asterisk (^*^) indicated a significant model improvement by using height profile rather than both maximum and mean heights (see Table S15 for detailed *F*-test results). In addition, Pearson's correlation coefficients were calculated for all univariate traits (see Figure S5).

In general, static traits had some value (*R*^2^ = 0.12–0.71) for predicting cotton fiber yield (Figure 11A). Among univariate traits, multi-dimensional traits (e.g., projected canopy area and volume) considerably outperformed one-dimensional traits such as canopy height and width, presumably because multi-dimensional traits could depict canopy size more completely. For instance, genotypes GA2011158 and GA2009037 had similar canopy height but different fiber yield, resulting in a low correlation between canopy height and fiber yield. However, the two genotypes had obvious differences in projected canopy area and canopy volume, with improved correlation between the morphological traits and fiber yield. This indicated the usefulness of extracting multi-dimensional traits using advanced 3D imaging techniques. Compared with univariate traits (the maximum and mean canopy height), multivariate traits (cumulative height profile) showed a significant improvement in yield prediction on most days (see detailed *F*-test results in Table S15). This was because cumulative height profiles intrinsically incorporated spatial distribution: high percentile ranks represented height information in the middle of the canopy, whereas low percentile ranks represented the borders. The capability of extracting multivariate traits was an advantage of using 3D imaging modalities. Most static traits achieved the highest correlation with fiber yield after the canopy development stage (DAP 67), and then the correlation decreased slightly during the rest of the growing season. In the growing season that was studied, the best period to use morphological traits for yield prediction was the transition period between canopy development and flowering and boll development stages. Canopy size during the transition period reached the maximum size and reasonably represented the potential of flower and boll development. However, mean canopy width and projected canopy area achieved the highest correlation at later dates (DAP 95 and DAP 102) because all genotypes kept expanding until DAP 95 and most of the expansion was due to the development of sympodial (fruiting) branches.

Compared with static traits, dynamic traits (growth rates) demonstrated less capability for yield prediction, but multi-dimensional traits that could comprehensively quantify canopy size still outperformed one-dimensional traits (Figure 11B). This further supported the advantage of using 3D imaging for measuring morphological traits. In contrast to static traits, growth rates were more informative for yield prediction in early stages (early periods in canopy development stage) rather than late stages. This may have been because all four genotypes were short season cotton and had similar overall growth trends. In early stages, growth rates were positive, reflecting canopy development, and were well correlated with the final vegetative structure and productive capacity. However, in late stages, growth rates were negative, reflecting leaf and plant senescence, and were not well correlated with fiber yield.

## 4. Discussion

Consumer-grade RGB-D image-based measurement of plant canopies in field conditions could lower the cost and increase the use of imaging techniques in phenotyping, benefitting the plant science community. The entire system used in the present study cost $70,500, including an RGB-D camera ($200), high-clearance tractor platform ($60,000), RTK-GPS ($8,000), power unit ($300), and rugged laptop ($2,000). This is still a considerable investment, but can be reduced. The tractor platform can be replaced with a low-cost pushcart (usually less than $2,000) if experiments are less than a hectare (Bai et al., 2016). In addition, a pushcart system allows for “stop-measure-go” mode, in which image acquisition locations can be predefined and manually controlled, and thus RTK-GPS is not required. However, these low-cost solutions decrease the efficiency and throughput of data collection. The consumer-grade RGB-D camera is an inexpensive 3D sensing solution for plant phenotyping, but building a phenotyping system requires comprehensive consideration of balance between research budget, experimental scale, and data collection and processing throughput.

The processing algorithms reported here can accurately extract morphological traits of plant canopies at the plot level, providing useful information for genomic studies and/or breeding programs. However, we note two limitations of the proposed approach. First, the Kinect v2 camera cannot be directly used under strong illumination (e.g., during midday in the field), so a mechanical structure is required to provide shade. Second, the reconstruction step currently relies on functions provided by Kinect v2 SDK, and must be performed on a computer running on an operating system of Windows 8 (or later) with connection to the Kinect v2 camera. This may significantly decrease the post-processing throughput, because cloud computing services usually run on remote Unix/Linux systems and users cannot connect hardware components to them while processing. This limitation could be addressed with third party libraries or user-performed registration between depth and color images (Kim et al., 2015). We acknowledge that the developed method was tested in a 1-year experiment, and altered conditions may change results. For example, predictors of yield were most effective near the end of canopy development in this experiment—however, dramatic differences in growing conditions (such as drought or cold) could change that in other years. Additional experiments might also consider different degrees of replication—here, a total of 32 replicates per genotype were used, which is considerably more than the number that is generally used in genetics/genomics studies. Variability among these samples provides a basis for estimating the minimal number of samples that might be used to discern differences between treatments of pre-determined magnitude, which is important in experiments involving large numbers of genotypes such as breeding programs. Based on theoretical calculation (Cochran and Cox, 1992), most static traits required three replicates, whereas most dynamic traits needed more than ten replicates (see Figure S6). This is because the four genotypes used in this study are very similar in growth patterns, requiring a high number of replicates to increase the statistical power for genotype differentiation. However, the minimal number of replicates could be reduced for growth rates in experiments involving genotypes with distinctive growth patterns (e.g., wild and elite cotton germplasm lines).

Two important findings were observed for the extracted traits. Firstly, static and dynamic traits showed the highest correlation with fiber yield in different periods; dynamic traits were informative in early canopy development stages, whereas most static traits were useful in the transition period between canopy development and flower and boll development. Canopy size (canopy height, width, projected area, and volume) remained relatively constant near its peak for a period after canopy development, indicating to some extent the capability to develop flowers and bolls. However, growth rates were negative in late stages, reflecting plant senescence as resources were redirected to the maturing bolls. Secondly, multivariate traits consistently showed better yield prediction than univariate traits, presumably because the multivariate traits intrinsically incorporated information such as the spatial variation of canopy height. Several previous studies reported methods to find the best percentile of canopy height to use to increase the accuracy of yield prediction (Friedli et al., 2016; Weiss and Baret, 2017). However, according to our results, it may be more efficient to explore the use of multivariate traits to predict yield. It is noteworthy that environment is an important factor, and analysis of these parameters in different growing seasons with various environments may yield different results. These differences may help to better explain interactions between genotype and environment (G × E), and make breeding programs more effective.

## 5. Conclusions

The 3D imaging system and data processing algorithms described here provided an inexpensive solution to accurately quantify cotton canopy size in field conditions. The extracted morphological traits showed potential for yield prediction. Multidimensional traits (e.g., projected canopy area and volume) and multivariate traits (e.g., cumulative height profile) were better yield predictors than traditional univariate traits, confirming the advantage of using 3D imaging modalities. Early canopy development stages were the best period in which to use dynamic traits for yield prediction, whereas late canopy development stages were the best period in which to use static traits. Future studies will be focused on improving the data processing throughput (via methods such as parallel computing) and extracting new multivariate traits for canopy size quantification.

## Author contributions

YJ, CL, and AP conceived the main concept of the study; YJ, JR, SS, RX, AP, and CL designed the experiment and performed field data collection; YJ developed the data processing pipeline; YJ, CL, AP, SS, and RX analyzed the data; AP and CL provided plant materials, instruments, and computing resources used in the study; YJ, CL, and AP contributed to writing the manuscript. All authors reviewed the manuscript.

### Conflict of interest statement

The authors declare that the research was conducted in the absence of any commercial or financial relationships that could be construed as a potential conflict of interest.
