# Supplementary material for: Quantitative Analysis of Cotton Canopy Size in Field Conditions Using a Consumer-Grade RGB-D Camera
Source: Front Plant Sci. 2018 Jan 30;8:2233. doi: 10.3389/fpls.2017.02233 (PMC5797632; doi:10.3389/fpls.2017.02233)
Supplement: Supplementary file 1 [file Presentation1.pdf]

Supplementary Material: Quantitative analysis of cotton canopy size in field conditions using a consumer-grade RGB-D camera

Yu Jiang [1], Changying Li [1,*], Andrew Paterson [2,3], Shangpeng Sun [1], Rui Xu [1], and Jon Robertson [2]

*Correspondence:
Corresponding author at 712F Boyd Graduate Studies, 200 D. W. Brooks Drive, The University of Georgia, Athens, Georgia, 30602, United States of America. Phone: (706) 542-4696; Fax: (706) 542-2475.
cyli@uga.edu

1 SUPPLEMENTARY TABLES AND FIGURES

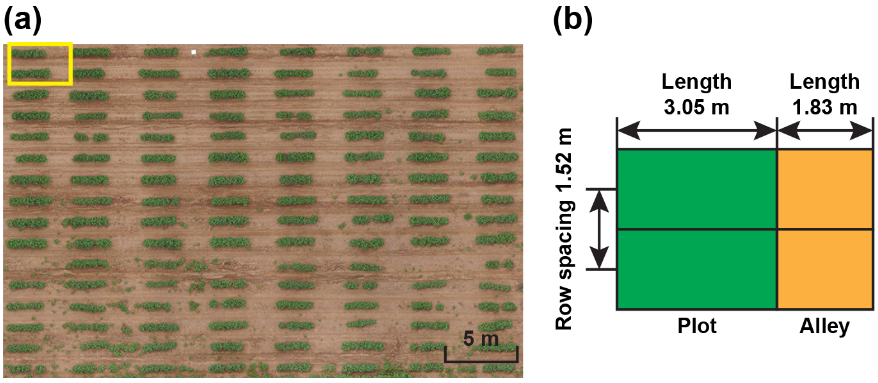

Figure S1: Experimental field used in this study: (a) aerial image of the entire field and (b) plot dimensions.

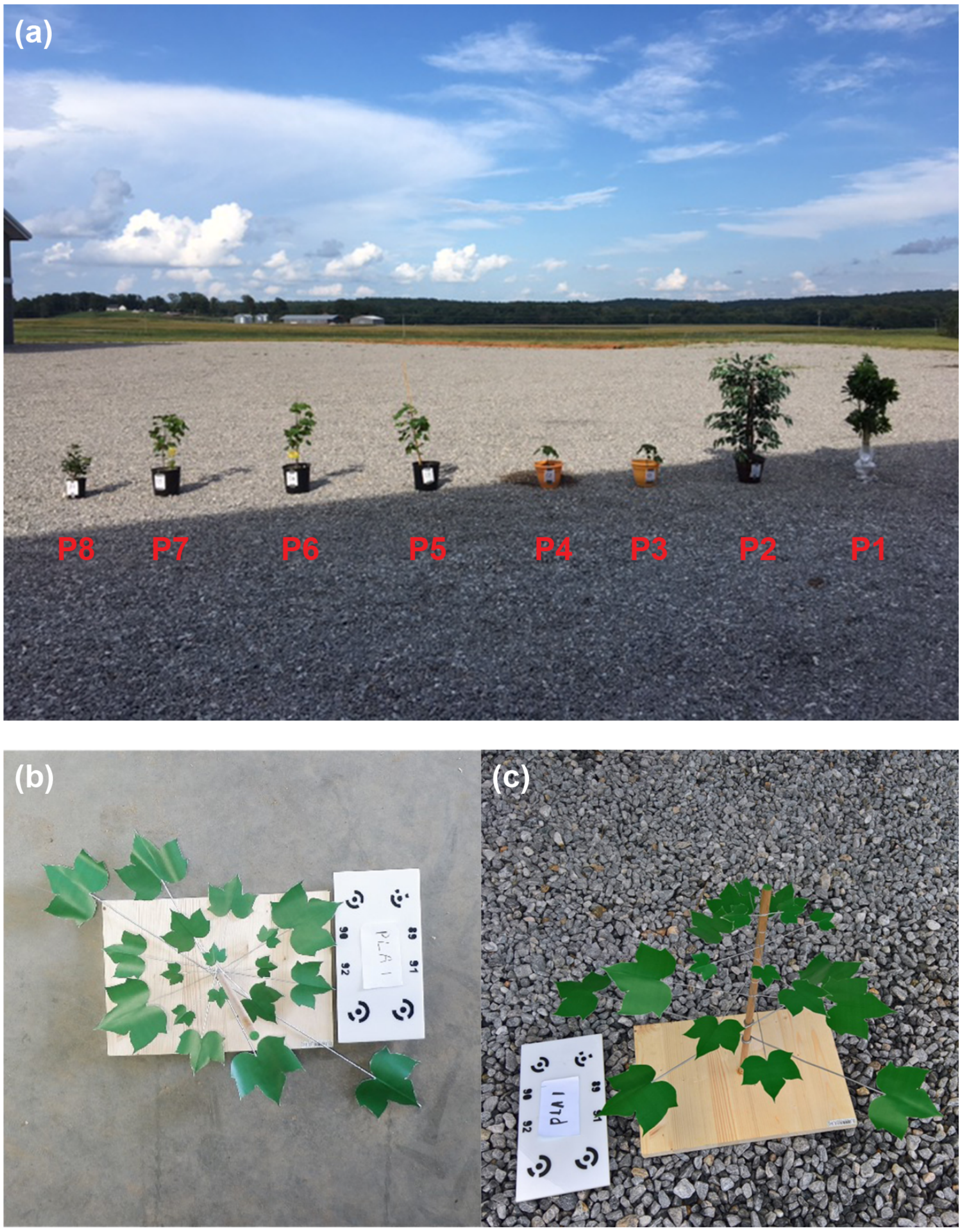

Figure S2: Potted and artificial plants used for accuracy validation: (a) 8 potted plants, (b) the artificial plant with all leaves laid on, and (c) the artificial plant with leaves lifted at various heights

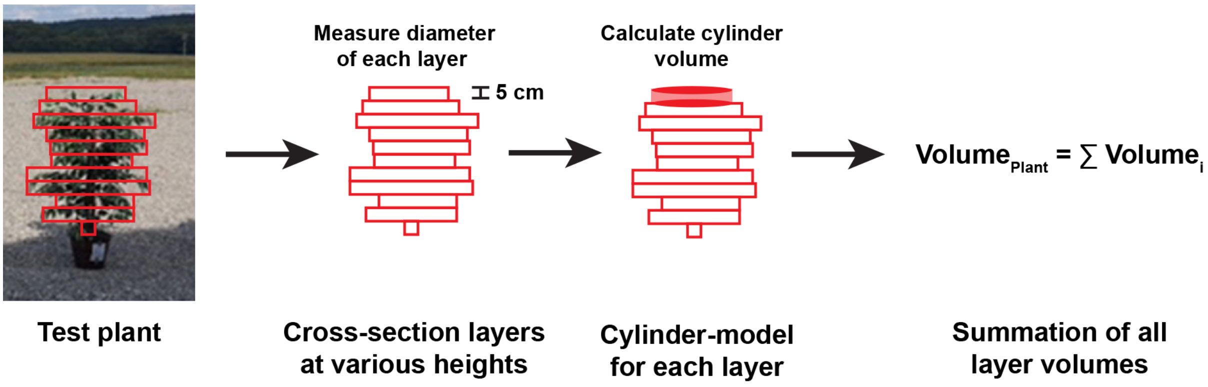

Figure S3: Diagram of using the tree volume estimation protocol to measure reference volume of potted plants.

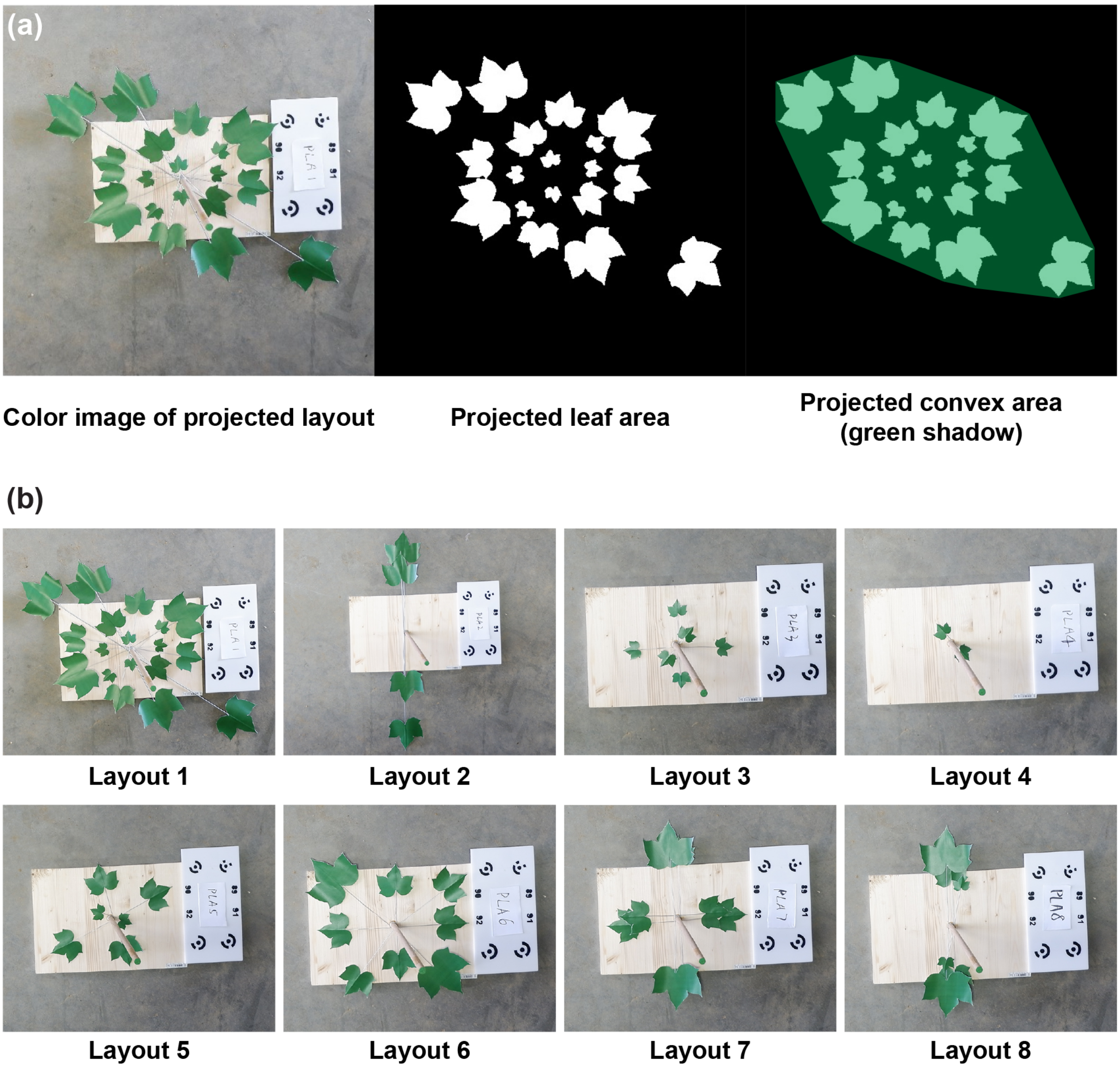

Figure S4: The artificial plant with various layouts for assessing the accuracy of measuring projected leaf/convex area: (a) an example of color image taken using a DSLR camera from the top view, masked (white pixels) area represented projected leaf area, and green shadowed area represented the projected convex area; and (b) 8 layouts used in the present study for projected leaf/convex area validation

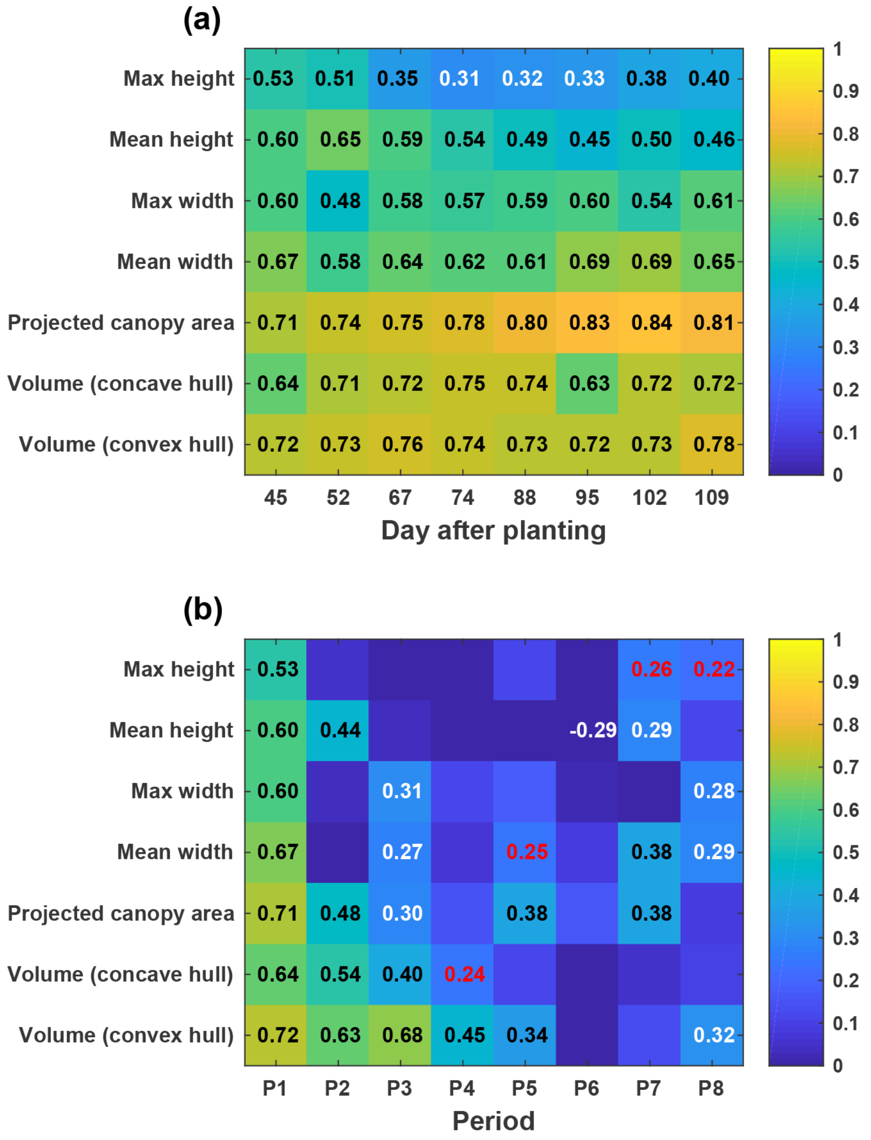

Figure S5: Pearson's correlation coefficients (r) between extracted traits and cotton fiber yield: (a) static traits and fiber yield and (b) dynamic traits and fiber yield. Black, white, and red colors indicated statistical significance of '<0.001', '<0.01', and '<0.05', respectively. Insignificant r values were not shown in blocks. P1 was the period from the day of planting to DAP 45, and P2 to P8 were the periods between two consecutive data collection dates.

Table S1. Statistical differences in maximum canopy height on eight days among genotypes. Different letters denoted statistical differences, and the alphabetical order indicated the value of growth rates in descending order.

Genotype	D45	D52	D67	D74	D88	D95	D102	D109
GA2011158	A	A	A	A	A	A	A	A
GA2009037	A	A	A	A	A	A	A	A
GA2010074	B	B	B	B	B	B	B	B
Commercial	B	B	C	C	C	C	C	C

Table S2. Statistical differences in mean canopy height on eight days among genotypes. Different letters denoted statistical differences, and the alphabetical order indicated the value of growth rates in descending order.

Genotype	D45	D52	D67	D74	D88	D95	D102	D109
GA2011158	A	A	A	A	A	A	A	A
GA2009037	A	A	A	A	AB	AB	AB	AB
GA2010074	A	A	B	B	B	B	B	B
Commercial	B	B	C	C	C	C	C	C

Table S3. Statistical differences in maximum canopy width on eight days among genotypes. Different letters denoted statistical differences, and the alphabetical order indicated the value of growth rates in descending order.

Genotype	D45	D52	D67	D74	D88	D95	D102	D109
GA2011158	A	A	A	A	A	A	A	A
GA2009037	B	A	B	B	B	B	B	B
GA2010074	A	A	AB	B	B	AB	B	AB
Commercial	A	A	B	B	B	B	B	B

Table S4. Statistical differences in mean canopy width on eight days among genotypes. Different letters denoted statistical differences, and the alphabetical order indicated the value of growth rates in descending order.

Genotype	D45	D52	D67	D74	D88	D95	D102	D109
GA2011158	A	A	A	A	A	A	A	A
GA2009037	A	AB	B	B	B	B	B	B
GA2010074	A	B	B	B	B	A	B	A
Commercial	A	B	B	B	B	B	B	A

Table S5. Statistical differences in projected leaf area on eight days among genotypes. Different letters denoted statistical differences, and the alphabetical order indicated the value of growth rates in descending order.

Genotype	D45	D52	D67	D74	D88	D95	D102	D109
GA2011158	A	A	A	A	A	A	A	A
GA2009037	B	A	B	B	B	C	C	B
GA2010074	A	A	AB	AB	AB	AB	AB	A
Commercial	AB	A	AB	B	B	BC	BC	A

Table S6. Statistical differences in concave hull volume on eight days among genotypes. Different letters denoted statistical differences, and the alphabetical order indicated the value of growth rates in descending order.

Genotype	D45	D52	D67	D74	D88	D95	D102	D109
GA2011158	A	A	A	A	A	A	A	A
GA2009037	B	A	AB	AB	A	B	B	B
GA2010074	AB	A	AB	B	A	AB	AB	A
Commercial	B	A	B	B	B	C	B	B

Table S7. Statistical differences in convex hull volume on eight days among genotypes. Different letters denoted statistical differences, and the alphabetical order indicated the value of growth rates in descending order.

Genotype	D45	D52	D67	D74	D88	D95	D102	D109
GA2011158	A	A	A	A	A	A	A	A
GA2009037	B	AB	B	B	B	B	B	B
GA2010074	AB	AB	B	B	B	B	B	B
Commercial	B	B	B	C	C	C	C	B

Table S8. Statistical difference in growth rates of maximum canopy height among genotypes. Different letters denoted statistical differences, and the alphabetical order indicated the value of growth rates in descending order. P1 to P5 were the periods from the day of planting to DAP 45, DAP 45 to DAP 52, DAP 52 to DAP 67, DAP 67 to DAP 74, and DAP 74 to DAP 88, respectively.

Genotype	P1	P2	P3	P4	P5
GA2011158	A	A	A	A	A
GA2009037	A	A	A	A	A
GA2010074	B	A	B	A	A
Commercial	B	B	B	B	A

Table S9. Statistical difference in growth rates of mean canopy height among genotypes. Different letters denoted statistical differences, and the alphabetical order indicated the value of growth rates in descending order. P1 to P5 were the periods from the day of planting to DAP 45, DAP 45 to DAP 52, DAP 52 to DAP 67, DAP 67 to DAP 74, and DAP 74 to DAP 88, respectively.

Genotype	P1	P2	P3	P4	P5
GA2011158	A	A	A	A	A
GA2009037	A	A	A	A	A
GA2010074	A	A	B	A	A
Commercial	B	B	B	B	B

Table S10. Statistical difference in growth rates of maximum canopy width among genotypes. Different letters denoted statistical differences, and the alphabetical order indicated the value of growth rates in descending order. P1 to P5 were the periods from the day of planting to DAP 45, DAP 45 to DAP 52, DAP 52 to DAP 67, DAP 67 to DAP 74, and DAP 74 to DAP 88, respectively.

Genotype	P1	P2	P3	P4	P5
GA2011158	A	A	A	A	A
GA2009037	B	A	A	A	A
GA2010074	A	B	A	A	A
Commercial	A	A	A	B	A

Table S11. Statistical difference in growth rates of mean canopy width among genotypes. Different letters denoted statistical differences, and the alphabetical order indicated the value of growth rates in descending order. P1 to P5 were the periods from the day of planting to DAP 45, DAP 45 to DAP 52, DAP 52 to DAP 67, DAP 67 to DAP 74, and DAP 74 to DAP 88, respectively.

Genotype	P1	P2	P3	P4	P5
GA2011158	A	A	A	A	A
GA2009037	A	A	A	AB	B
GA2010074	A	B	A	A	A
Commercial	A	B	A	B	A

Table S12. Statistical difference in growth rates of projected leaf area among genotypes. Different letters denoted statistical differences, and the alphabetical order indicated the value of growth rates in descending order. P1 to P5 were the periods from the day of planting to DAP 45, DAP 45 to DAP 52, DAP 52 to DAP 67, DAP 67 to DAP 74, and DAP 74 to DAP 88, respectively.

Genotype	P1	P2	P3	P4	P5
GA2011158	A	A	A	A	A
GA2009037	B	A	A	AB	A
GA2010074	A	A	A	A	A
Commercial	B	A	A	B	A

Table S13. Statistical difference in growth rates of concave hull volume among genotypes. Different letters denoted statistical differences, and the alphabetical order indicated the value of growth rates in descending order. P1 to P5 were the periods from the day of planting to DAP 45, DAP 45 to DAP 52, DAP 52 to DAP 67, DAP 67 to DAP 74, and DAP 74 to DAP 88, respectively.

Genotype	P1	P2	P3	P4	P5
GA2011158	A	A	A	A	A
GA2009037	B	A	A	A	A
GA2010074	AB	A	B	A	A
Commercial	B	A	A	B	A

Table S14. Statistical difference in growth rates of convex hull volume among genotypes. Different letters denoted statistical differences, and the alphabetical order indicated the value of growth rates in descending order. P1 to P5 were the periods from the day of planting to DAP 45, DAP 45 to DAP 52, DAP 52 to DAP 67, DAP 67 to DAP 74, and DAP 74 to DAP 88, respectively.

Genotype	P1	P2	P3	P4	P5
GA2011158	A	A	A	A	A
GA2009037	B	A	B	A	A
GA2010074	AB	A	B	A	AB
Commercial	B	A	B	B	B

Table S15. Results of the F-test between regression models established using a multivariate trait (cumulative height profile) and a univariate trait (either the maximum or mean canopy height).

Date (day after planting)	Difference between regression models established using cumulative height profile and maximum canopy height		Difference between regression models established using cumulative height profile and mean canopy height	
	F-value	P-value	F-value	P-value
28 July 2016 (DAP 45)	1.75	0.048	1.15	0.32
4 August 2016 (DAP 52)	2.52	0.003	1.04	0.43
19 August 2016 (DAP 67)	6.19	<0.001	3.5	<0.001
26 August 2016 (DAP 74)	3.65	<0.001	2	0.02
9 September 2016 (DAP 88)	3.21	<0.001	2.09	0.014
16 September 2016 (DAP 95)	3.25	<0.001	2.52	0.003
23 September 2016 (DAP 102)	2.46	0.004	1.71	0.057
30 September 2016 (DAP 109)	1.48	0.12	1.18	0.29

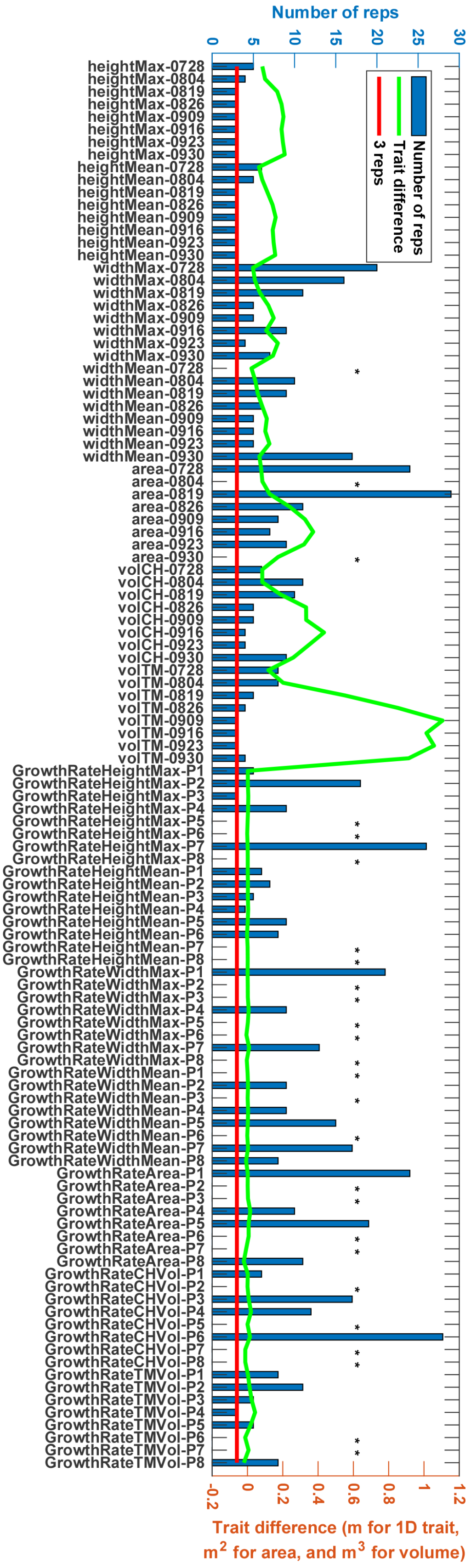

Figure S6: Results of the estimated minimum number of replicates for each extracted trait which can provide significantly differences between the GA2011158 and Americot Conventional (commercial cultivar). The number of replicates equals -1, if its estimated value exceeds 30.

Generally, three replicates provided adequate statistical power to differentiate GA2011158 from Americot conventional by using most static traits, especially in late growth stages (e.g. after 9 August 2016, which was 67 days after planting (DAP 67)). However, growth rates (dynamic changes of static traits) required more replicates to discern the two genotypes. This was because GA2011158 is an elite breeding line for yield production, and thus having a similar growth pattern with commercial cultivars.
